# Supplementary material for: Autophagy is dispensable in germline stem cells but is required in the cap cells for their maintenance in the Drosophila ovarian niche
Source: Stem Cell Reports. 2025 Nov 13;20(12):102712. doi: 10.1016/j.stemcr.2025.102712 (PMC12744849; doi:10.1016/j.stemcr.2025.102712)

Stem Cell Reports, Volume *20*

Supplemental Information

Autophagy is dispensable in germline stem cells but is required in the cap cells for their maintenance in the *Drosophila* ovarian niche

Kiran Suhas Nilangekar and Bhupendra V. Shravage

Supplementary text

Materials and methods

Fly maintenance

Flies were maintained at standard conditions; 25℃, 60-70% relative humidity, 12-12-hour light-dark cycle. Fly food composition per liter of food; 80g sugar, 75g corn flour, 30g yeast, 30g malt extract, 10g agar, 0.12% methyl benzoate, 0.4% propionic acid and 0.08% orthophosphoric acid. For setting crosses, 0–5-day old males and virgin females were used. Until dissection, the flies of desired genotypes were housed as 15 females and 7-10 males in a vial which were flipped every two to three days to fresh food vials supplemented with dry yeast pellets. Two days prior to dissection, the ovaries were fattened by transferring the flies, each day on fresh food vials supplemented with dry yeast pellets. Diethyl ether vapors were used to anesthetize flies. For chloroquine treatment, a stock solution of 50 mg/ml chloroquine in water was prepared from chloroquine diphosphate salt (Sigma Aldrich, C6628) for experiments in Figure 1A and Figure S1A. The 50 mg/ml chloroquine stock solution was added to fly food to a final concentration of 3 mg/ml when the food cooled down to 50-60℃ during preparation. Flies from treated and untreated sets were first fed on food supplemented with yeast pellets for two days before being subjected to treatment. The flies for chloroquine treatment were transferred to fresh chloroquine containing food vials for two days while the untreated flies were transferred to fresh vials containing only normal fly food. For experiments in Figure S1C and D, a stock solution close to 50 mg/ml was prepared from chloroquine tablets (Chloroquine phosphate IP 500 mg, Lariago®-DS, Ipca Laboratories ltd.), the tablets were dissolved in water and the insoluble excipients were filtered through 0.22 µm filter. For these experiments, freshly eclosed flies were fed on food supplemented with yeast pellets for three days prior treatment and subsequently transferred to fresh vials having either chloroquine containing food (3 mg/ml) or only normal fly food, for three days.

Recombination of Atg5 mutant and FRT19A and its validation

The *Atg5*^{5cc5} mutant was first crossed to *isoFRT19A* and the virgin female progeny was crossed to *FM7i* males. After a day of setting the cross, the flies were transferred to vials containing 500 µg/ml geneticin (G418) (Gibco, 11811-023) for egg laying. G418 was used to screen the FRT19A positive recombinants since the FRT19A cassette has neomycin as a selection marker. All the surviving virgin female progeny developed from the G418 vials were crossed to *FM7i* males in multiple single pair crosses. Males from the progeny of each of the single pair crosses were used to perform single fly PCR to confirm the presence of mutation using primers that specifically bind in the region of *Atg5* which was

deleted in *Atg5*^{5cc5} (forward primer: 5'GCACTACATGTCCTGCCTGA, reverse primer: 5'AGATTCGCAGGGGAATGTTT).

FLP-FRT based GSC clone generation and GSC retention assay

Large cross of *Atg5*^{5cc5} *FRT19A* with *whsFLP, Ubi-RFP FRT19A* for Atg5 mutant GSC clones and another cross of *isoFRT19A* with *whsFLP, Ubi-RFP FRT19A* for control GSC clones were set in multiple vials. The crosses were transferred to a set of fresh vials supplemented with yeast paste and removed after six-seven hours to obtain synchronized egg lay. The vials were subjected to heat shock for four consecutive days on seventh through tenth day after egg lay i.e., during pupal development. Heat shocks were applied for 50 minutes twice a day, six-eight hours apart, at 37℃ in a water bath. Only the flies which eclosed after the complete heat shock regime were collected. Therefore, the days-post-eclosion is the same as days-post-heat-shock. Females of the appropriate genotype were collected and aged until the stipulated timepoints. For each time-point, 8-10 of the collected females were dissected two, nine and nineteen days-post-heat-shock and immunostained for hts (1B1) and Lamin C for identification and quantification of RFP^{+} and RFP^{-} GSCs.

For fed/starved treatment in the GSC clone retention assay, 'complete starvation vials' containing 0.8% agar were prepared; 0.8% agar (w/v) was dissolved in water by heating, and methyl benzoate to a final concentration of 0.12% was added when the solution cooled to 50-60℃. Flies from all sets for this experiment were initially fed for three consecutive days post-eclosion by transferring them to fresh vials containing food supplemented with yeast pellets. At this stage, a subset of control and *Atg5* mutant flies were dissected to quantify the GSC clones' frequency before the fed/starved treatment. Consequently, sets of flies were transferred to either complete starvation vials or vials with food supplemented with yeast pellets for four consecutive days for the fed/starved treatment and then dissected. All sets were immunostained for hts (1B1) and Lamin C for identification and quantification of RFP^{+} and RFP^{-} GSCs.

Immunostaining of Ref(2)P in *Atg5* mutant GSCs was performed in two experiments. First using *Ubi-RFP FRT19A* where the progeny was heat shocked in the adult stage ten days post eclosion for three consecutive days in the regime stated above, and dissections were performed two days after the last heat shock. Second where the immunostaining was performed two-days post eclosion after pupal heat shock. The same procedure was repeated while using *His2AvGFP FRT19A* and the phenotype could be replicated (data not shown).

Immunostaining

Flies were briefly anesthetized and dissected in Grace's medium (Gibco, 11667-037). Ovaries were extracted and non-vitellogenic region of the ovarioles were partially teased apart using minutien pins. The ovaries were then transferred to 0.5 ml tubes with the aid of a cut-tip passivated with bovine serum albumin (BSA) (MP Biomedicals, 199897). The ovaries were fixed with 350 µl 4% paraformaldehyde (Sigma Aldrich, P6148) in 1xPBS (phosphate-buffered saline) pH 7.4 for 15 minutes at room temperature with the gentle nutation. All the following steps are performed with gentle nutation 15-20 RPM. The fixative was washed off with three washes of 400 µl 0.1% PBTx (0.1% triton-X-100{SRL, 64518} in 1xPBS) for five minutes each. Blocking was performed in 300 µl of 0.5% BSA in 1% PBTx for one hour at room temperature. The sample was incubated with at least 100 µl of the appropriate primary antibody solution in which the antibody was diluted in 0.3% PBTx containing 0.5% BSA. The incubation with primary antibody solution was performed at 4℃ overnight with gentle nutation 5 RPM. The primary antibody was washed off with 400 µl 0.1% PBTx for 15 minutes at room temperature. Following which the samples were again blocked for secondary antibody staining with 400 µl of 10% normal goat serum (NGS) (MP Biomedicals, 2939149) in 0.1% PBTx for two hours at room temperature. The secondary antibody diluted in the 10% NGS solution in 0.1% PBTx was incubated with the samples for two hours at room temperature. Three washes of 15 minutes each with 400 µl of 0.1% PBTx were performed at room temperature. For DAPI staining, the samples were incubated in 1 µg/ml DAPI in 0.1% PBTx solution for 10 minutes at room temperature and consequently washed off twice for five minutes each with 400 µl 0.1% PBTx. The samples were stored at 4℃ until mounting. All the PBTx solution was removed carefully and mounting medium SlowFade Glass mountant (Invitrogen, S36917) was added. All the ovaries were transferred onto a slide along with the mounting medium. In order to obtain flat mounting of the germarium, the region of the ovarioles which have the string of pre-vitellogenic stages and germarium at the tip were separated using minutien pins and the remaining large part of the ovaries was picked and removed from the slide. For optimal Confocal microscopy, ~170 µm thick no.1 coverslips were used. Nail varnish was used to seal the slide. The slides were stored at 4°C protected from light until microscopy. All the anti-pMad staining were executed with a same workflow for consistency across timepoints.

The following primary antibodies were used with the mentioned dilutions; anti-Cathepsin L (Abcam, ab58991; 1:400), anti-Ref(2)P (Abcam, ab178440; 1:1000), anti-Hts (DSHB, 1B1; 1:50), anti-Lamin C (DSHB, LC28.26; 1:50), anti-pMad (Abcam, ab52903; 1:50). The following secondary antibodies were used at 1:250 dilution; Goat anti-Rabbit Alexa Fluor 555 (Thermo Fisher Scientific, A21429), Goat anti-Rabbit Alexa Fluor 647 (Thermo Fisher Scientific, A21245) and Goat anti-Mouse Alexa Fluor 647 (Thermo Fisher Scientific, A21236).

TUNEL staining

In Situ Cell Death Detection Kit, TMR red (Roche, 12156792910) was used for TUNEL staining. The procedure was repeated exactly the same for all five time points. Ovaries were dissected and teased as described for immunostaining. All the following steps were performed with gentle nutation and the volumes indicated are for each sample. Fixation: 15 minutes at room temperature, 300 µl 4% paraformaldehyde in PBS pH 7.4. Followed by three washes of 5 minutes each at room temperature with 300 µl 0.1% PBTx for each. Subsequently, blocking and permeabilization: 300 µl of 1% PBTx containing 0.5% BSA, for one hour at room temperature. Followed by one wash with 400 µl 1x PBS for 2 minutes at room temperature. Total of 250 µl TUNEL reaction solution was prepared during each experiment, which comprised of 225 µl TUNEL TMR label solution and 25 µlTdT enzyme solution. 50 µl TUNEL reaction solution was used per sample. TUNEL reaction was performed by adding the TUNEL reaction solution to the sample and incubating for 2 hours at 37°C in a shaking incubator, 80 RPM and protected from light. All following steps were performed with samples protected from light. The samples were proceeded for immunostaining after blocking in 300 µl of 1% PBTx containing 0.5% BSA for 30 minutes at room temperature.

Confocal microscopy

Settings were identical across control versus test, a set of experiments or a session of imaging. All the microscopy performed on the Leica SP8 was done using 63x oil immersion objective NA=1.4. For all intensity and puncta count analyses performed on Leica SP8, the settings were as follows; resolution=1024x1024 pixels, scan speed=100 Hz, bit depth=8-bit. For obtaining z-stacks for GSC or cap cell counting; resolution=512x512 pixels, scan speed=400-600 Hz, bit depth=8-bit, z-step size=0.5 µm.

For the measurement of autophagy flux in GSCs, z-stacks spanning entire GSCs were acquired by visual confirmation from the DAPI (nucleus) channel. The z-step size was set to 0.4 µm which ensured that puncta were not missed out between steps. 63x oil immersion objective NA=1.4, 512x512 pixels at 0.05 µm per pixel resolution, scan speed=8, bit depth=16-bit.

For all GSC and cap cell counts, z-stacks of the tip of the germarium which covered the entire GSC niche were acquired by visual confirmation (z-step size=0.5 µm). While imaging for Ref(2)P puncta in the cap cells, the central plane of the cap cells was focused using the Lamin C channel. While imaging 3xmCherry-Atg8a in cap cells, the plane maximally covering the cluster of cap cells centrally was identified in the DAPI channel. The GSCs were identified by their location and size of the nucleus and the optical section across the central plane was acquired by focusing in the DAPI channel for the following

experiments; Ref(2)P in *Atg5* knockdown, *Atg5* overexpression, *Atg5* mutant, and pMad. Imaging settings for all puncta count experiments were exactly the same for all experimental conditions and the imaging of a set was completed in one imaging session. Imaging settings for all pMad intensity measurements were identical, except for 7-day timepoints where the frame accumulation was set to one, and the same was set to two for all others. The image acquisition was performed in the dynamic range where the non-tissue and tissue background were comparable among images of a set.

Image analysis

All the image analyses involving intensity measurement or puncta count were performed in ImageJ and Fiji. All steps performed for image analyses were exactly the same for all the control and test images in a data set. For GSC counts, *Atg5* mutant and control GSC clone counts, cap cell counts and TUNEL positive cap cell counts, LAS-X and Zeiss Zen 3.7 software were used to visualize and manually count the cells. GSCs were unequivocally identified by the following characteristics; their location, size of the nucleus, presence and orientation of the spectrosome. Cap cells were identified by their nuclear shape, location and Lamin C ring.

For autophagic flux determination in the GSCs, nosP-mCherry-Atg8a and Cathepsin L puncta within the GSCs were manually marked in the plane with maximal area and intensity. Additionally, colocalized mCherry-Atg8a and Cathepsin L puncta were also manually identified. Similarly, 3xmCherry-Atg8a puncta in the cap cells were manually quantified. For quantification of Ref(2)P puncta in the germaria, GSCs or cap cells; ROI was drawn to define either the germaria, GSCs or cap cells in the DAPI channel and the ROI was imported into the 'ROI manager'. The Ref(2)P channel was first subjected to the 'Subtract background' function with rolling ball radius 50 pixels or 35 pixels according to the optimal value for the data set. The resultant images were thresholded referring to the display under 'Max entropy' algorithm and then manually adjusted for each image such that the background was excluded but the true signal of the puncta did not erode. The thresholded images were then used for the function 'Analyze particles' in which the following parameters were set; Size (micron^2): 0.02-infinity or 0.03-infinity across a particular set according to the optimal value, Circularity: 0-1, from which the output was obtained as number and size of puncta within the marked ROI. pMad intensity in the GSCs was quantified in ImageJ by marking ROIs and recording the 'mean intensity'. The pMad intensities are plotted on different y-axes because each imaging was performed in different session upon attaining the timepoint.

Supplementary Figure S1: (A) Interleaved scatter plot showing the number of autophagic vesicles in *Atg5* overexpression (Atg5 OE) GSCs upon CQ treatment. n=20 GSCs per treatment. (B) Representative image of a cross-section of the central plane of the germarium showing eGFP fluorescence from eGFP-Atg5 (*Atg5* overexpression). eGFP fluorescence is visible in the GSC which marked by the dashed outline and bright puncta are visible in region 2. (C) Interleaved scatter plot showing the number of Ref(2)P puncta in control and *Atg5* overexpression (Atg5 OE) GSCs upon CQ treatment. n=21±1 GSCs per genotype per treatment. (D) Interleaved scatter plot showing Ref(2)P in the germarium upon CQ treatment. n=21±1 germaria per genotype per treatment. These experiments were performed twice independently, showing similar results. (E-E') Representative image showing Ref(2)P in the GSCs. The GSCs are marked by dotted outlines and the cap cells are marked by asterisks. Scale bar 10 µm. Interleaved scatter plots showing number (F) and size (G) of Ref(2)P puncta in GSCs. n=9 & 12 GSCs for control &*Atg5RNAi* respectively. Interleaved scatter plots showing the size (H) and number (I) of Ref(2)P puncta upon *Atg8a* knockdown in the cap cells and the same for size of puncta upon *Atg1* knockdown (J) in the cap cells. n=10±1 (*Atg8a RNAi*) and n=31±1 (*Atg1 RNAi*) cap cell planes/area per genotype. (K) List of all the Atgs tested for knockdown in the niche and the observation of Ref(2)P in cap cells. In all graphs blue line represents the average and error bars represent standard deviation. *$p<0.05$, **$p<0.01$, ****$p<0.0001$.

Supplementary Figure S2: (A) Graph showing change in the number of cells during aging. Average cap cell numbers are plotted as lines against the left y-axis and average GSC numbers are plotted as bars against the right y-axis. Cap cell numbers from the data set as indicated in Figure 3H with the addition of HhGal4/+ control. Error bars represent the standard error of the mean. (B) Stacked column graph showing distribution of the fraction of germaria with TUNEL-positive cap cells from the mentioned genotypes, out of all germaria assessed (cumulative) across age. n=80 germaria per genotype per time point for (A) and (B). Bar graphs showing the number of cap cells (C) and GSCs (D). Error bars represent standard deviation. Sample sizes are the number of germaria as indicated in the graphs. (E) Interleaved scatter plots showing pMad intensity in GSCs of the mentioned genotypes at the five mentioned timepoints. n=20±2 GSCs per genotype per time point.**$p<0.01$, ***$p<0.001$, ****$p<0.0001$.

Supplementary Figure S1

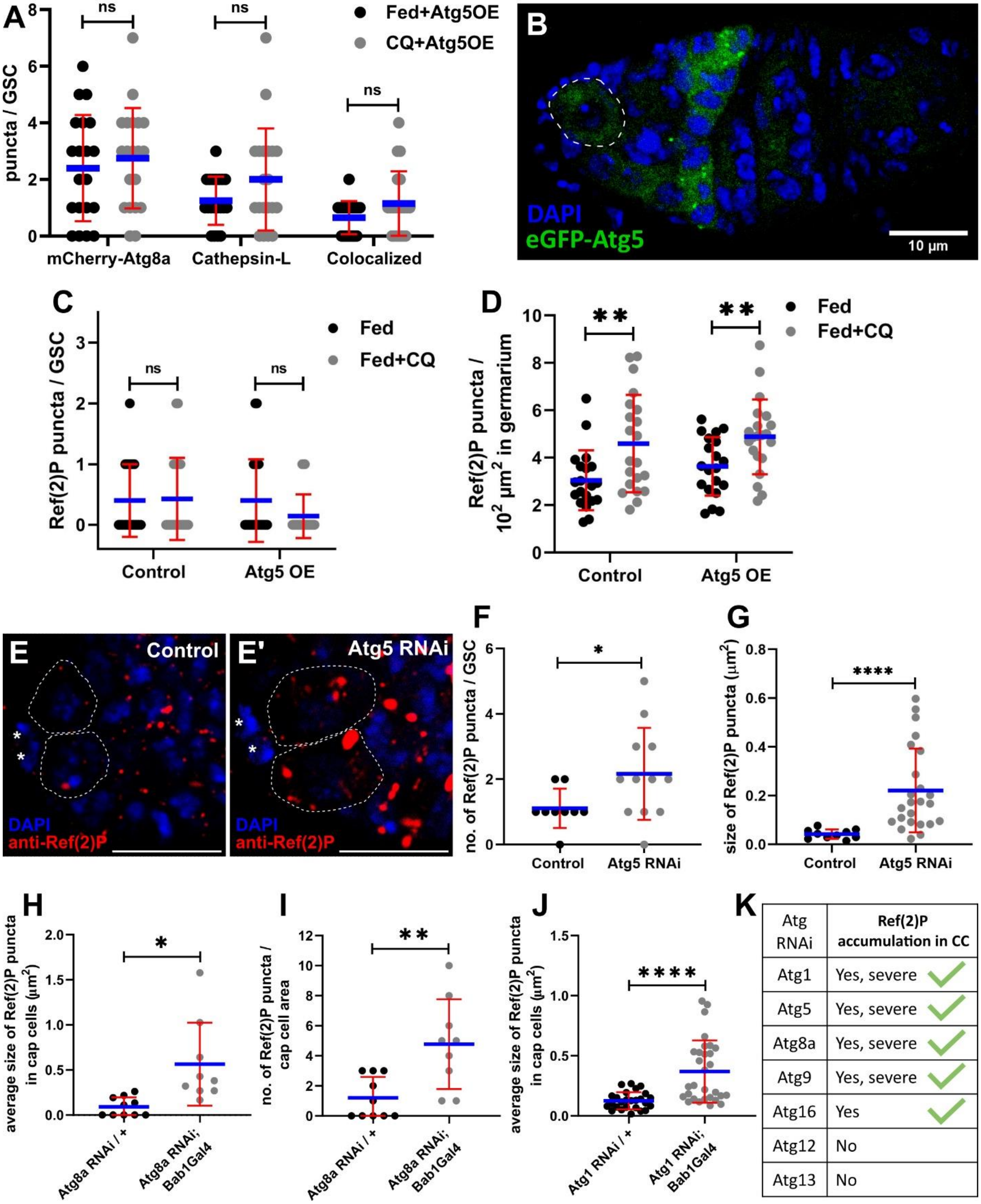

Atg RNAi	Ref(2)P accumulation in CC
Atg1	Yes, severe ✓
Atg5	Yes, severe ✓
Atg8a	Yes, severe ✓
Atg9	Yes, severe ✓
Atg16	Yes ✓
Atg12	No
Atg13	No

Supplementary Figure S2

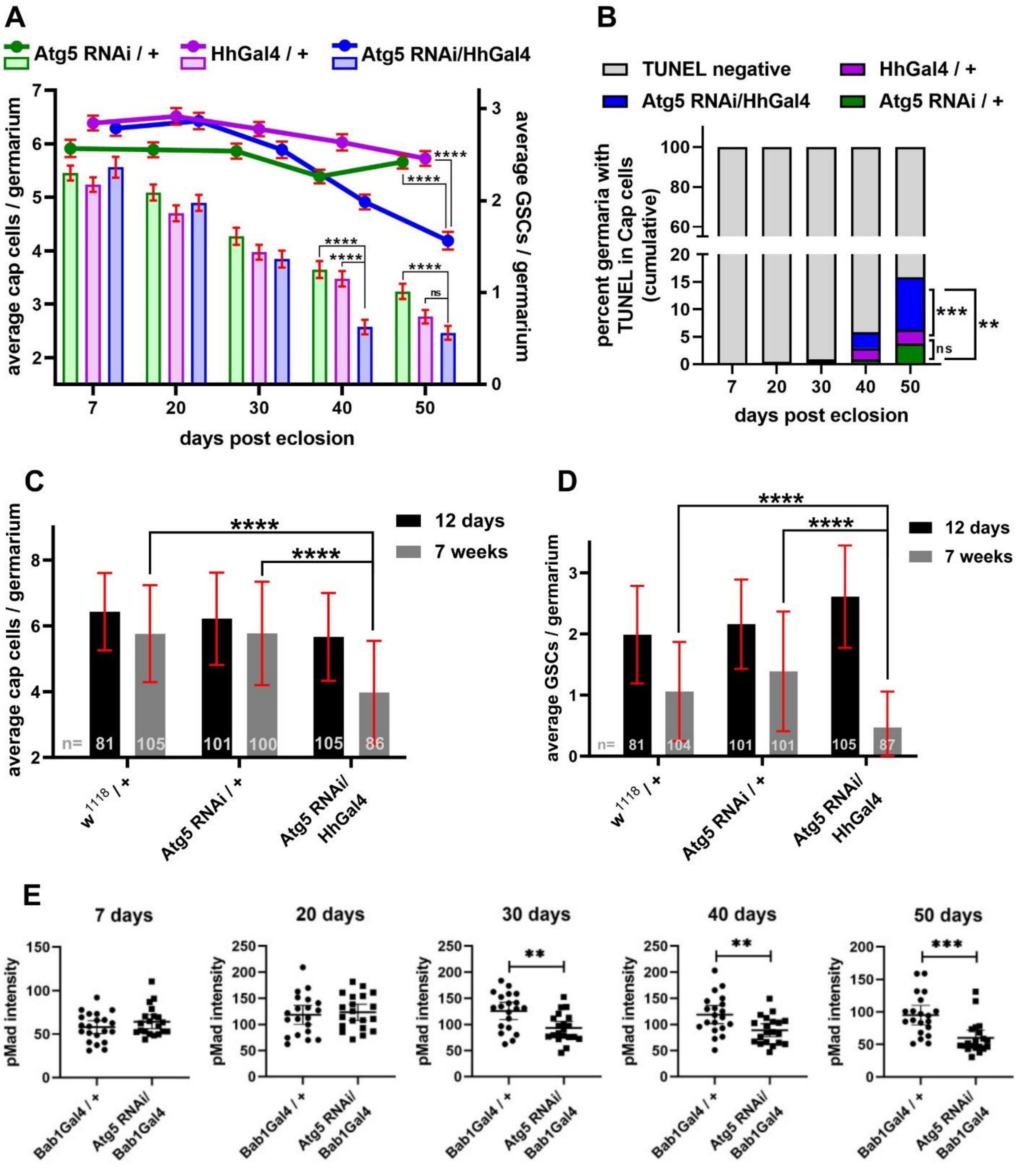

Supplement: Document S1. Figures S1 and S2 and supplemental materials and methods [file mmc1.pdf]
