# Supplementary material for: Autophagy is dispensable in germline stem cells but is required in the cap cells for their maintenance in the Drosophila ovarian niche
Source: Stem Cell Reports. 2025 Nov 13;20(12):102712. doi: 10.1016/j.stemcr.2025.102712 (PMC12744849; doi:10.1016/j.stemcr.2025.102712)

STEM CELL REPORTS
Report

OPEN ACCESS

Autophagy is dispensable in germline stem cells but is required in the cap cells for their maintenance in the *Drosophila* ovarian niche

Kiran Suhas Nilangekar[1,2] and Bhupendra V. Shravage[1,2,3,*]
[1]Developmental Biology Group, Agharkar Research Institute, Pune, India
[2]Department of Biotechnology, Savitribai Phule Pune University (SPPU), Pune, India
[3]Lead contact
*Correspondence: bvshravage@aripune.org
https://doi.org/10.1016/j.stemcr.2025.102712

SUMMARY

Autophagy is a cytoprotective mechanism responsible for the maintenance and long-term survival of various cell types, including stem cells. However, its role in the germline stem cell (GSC) niche remains unexplored. We demonstrate that autophagy flux in female *Drosophila* GSCs is low and dependent on the core autophagy gene, *Atg5*. However, the maintenance of $Atg5^{-/-}$ GSCs within the GSC niche was unaffected even under nutrient stress. In contrast, disruption of autophagy within the cap cells (niche cells) leads to the loss of both cap cells and GSCs during aging. Further, reduced autophagy in cap cells severely impairs the crucial GSC self-renewal signal mediated by BMP-pMad emanating from the cap cells at the onset of midlife. Autophagy was essential for the long-term survival of cap cells. Our study reveals a differential role for autophagy, which is dispensable in GSCs but necessary in niche cells, where it supports signaling and survival to maintain GSCs.

INTRODUCTION

Macroautophagy (autophagy hereafter) is a catabolic process that maintains cellular homeostasis by the sequestration and lysosome-mediated degradation of cytoplasmic material that is toxic or superfluous. Autophagy occurs constitutively at a basal level and is upregulated under stress conditions, including nutrient deprivation. Autophagy is an important factor that influences the aging and longevity of stem cells. The stem cell niche maintains the "stemness" of stem cells by activating pro-stemness cellular pathways and repressing differentiation. Stem cell niches replace dysfunctional cells within the tissues and organs but themselves age. However, the role of autophagy in the maintenance of stem cell niches throughout the lifespan is poorly understood.

Multiple Atg (autophagy-related) proteins form distinct complexes that regulate steps of autophagy (Klionsky et al., 2021). The Atg12-Atg5-Atg16 complex is essential for lipidation of Atg8 (LC3), which is crucial for the formation of mature autophagosomes. Knockdown (KD) of Atg5 blocks canonical autophagy (Chang et al., 2013; Klionsky et al., 2021; Rusten et al., 2004). $Atg5^{-/-}$ mice do not survive postnatally, while germline-specific knockout (KO) of *Atg5* disrupts sperm development and fertility in mice (Huang et al., 2021). Conditional KO of *Atg5* in murine neural stem cells showed impaired autophagy as observed by reduced LC3 lipidation, accumulation of p62, and dysfunctional mitochondria (Wang et al., 2016). Additionally, mutations in *ATG5* were found to cause ataxia in humans (Kim et al., 2016). Importantly, the ubiquitous overexpression of Atg5 in mice was sufficient to enhance autophagy levels and extend lifespan (Pyo et al., 2013). In *Drosophila*, basal autophagy is disrupted in both Atg5 KD and in *Atg5* null mutants (Kim et al., 2016; Scott et al., 2004). Given its essentiality for canonical autophagy, Atg5 is an ideal candidate for investigating autophagy and its role in the GSC niche.

Autophagy is crucial in adult stem cells for their self-renewal, differentiation, metabolism, and aging (Adelipour et al., 2022; Chen et al., 2018; Shravage and Turksen, 2023). In aged muscle stem cells and hematopoietic stem cells (HSCs), autophagy maintains quiescence and regenerative function (García-Prat et al., 2016; Ho et al., 2017). Autophagy is impaired in both aged murine and human muscle stem cells. Loss of autophagy drives stem cell senescence, a phenotype that can also be induced in young stem cells upon its disruption. Defective autophagy compromises proteostasis, perturbs mitochondrial quality control, and increases oxidative stress, resulting in reduced stem cell function and number. Notably, restoring autophagy reverses senescence and rejuvenates regenerative capacity. Interestingly, about one-third of aged HSCs maintain high autophagy, low metabolism, and robust regenerative potential, underscoring autophagy as a key determinant of stem cell fitness during aging (Ho et al., 2017).

It remains unclear whether autophagy supports niche cell maintenance and, in turn, indirectly affects GSCs. Our earlier work showed that Atg1 maintains GSCs by regulating mitochondrial dynamics, independent of canonical autophagy (Ayachit and Shravage, 2023). In *Drosophila*, loss of autophagy in male cyst stem cells (CySCs), a key niche component, disrupts their maintenance and causes GSC loss (Sênos Demarco et al., 2020). Previous studies

Published by Elsevier Inc. on behalf of International Society for Stem Cell Research.
This is an open access article under the CC BY license (http://creativecommons.org/licenses/by/4.0/).

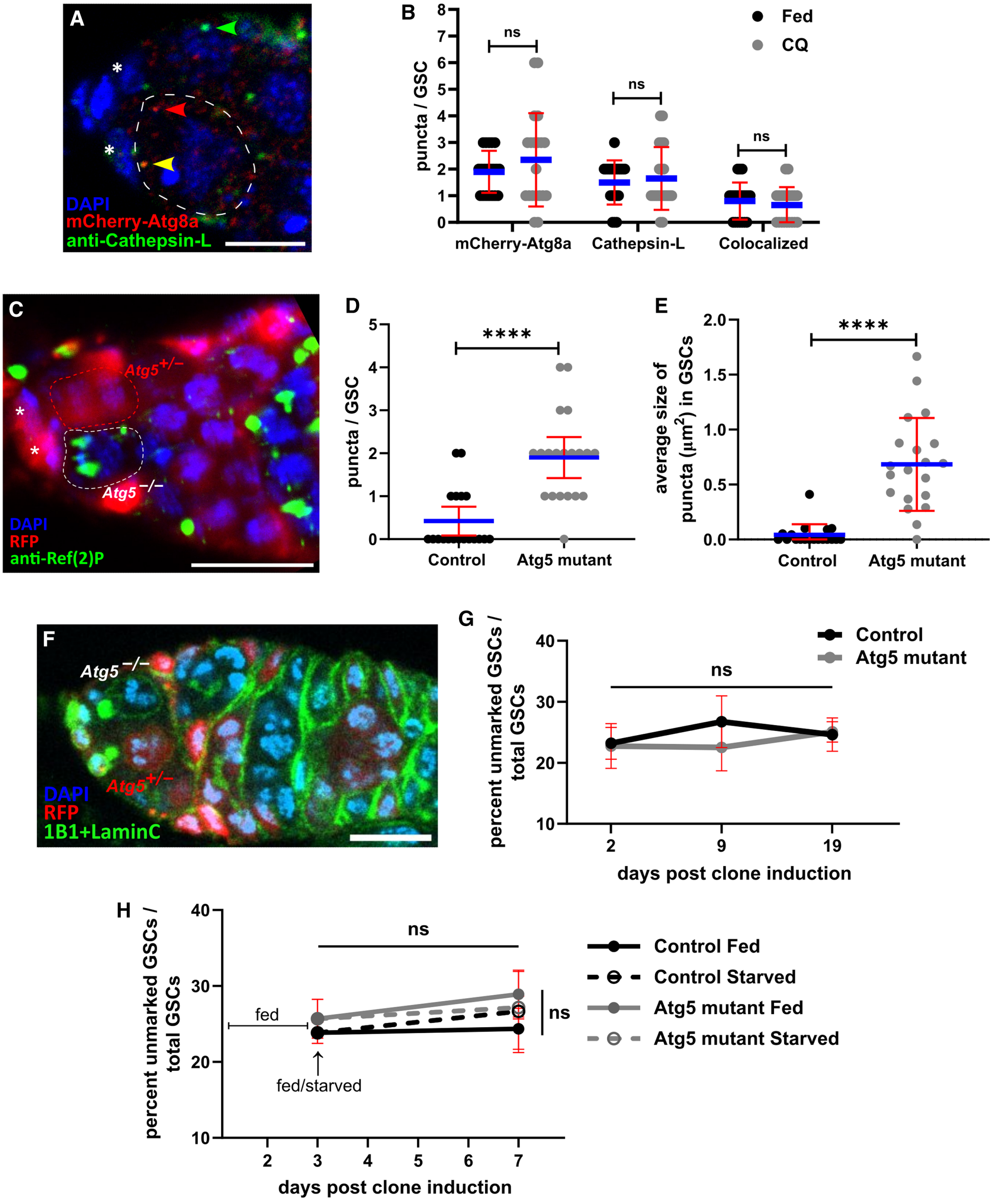

Figure 1. Autophagy in GSCs is low and dispensable for maintenance

(A) Representative image showing autophagic vesicles in GSCs. The GSC is marked by a dashed outline, and the cap cells are marked by asterisks. Puncta are indicated by colored arrowheads; mCherry-Atg8a/autophagosome (red), cathepsin L/lysosome (green), and colocalized punctum/autophagosome (yellow). Scale bar, 5 μm.

(B) Interleaved scatterplot showing the number of autophagic vesicles in control GSCs and CQ-treated GSCs. The blue line represents the average, and the error bars represent the SD. n = 20 GSCs per treatment.

(C) Representative image showing Ref(2)P in mosaic GSCs. *Atg5*-null (−/−) GSC is marked by a white dashed outline (RFP−), heterozygous (+/−) GSC is marked by a red dashed outline (RFP+), and cap cells are marked by asterisks. Scale bar, 10 μm. Interleaved scatterplot showing the number (D) and size (E) of Ref(2)P puncta in *Atg5* mutant GSCs. The blue line represents the average, and the error bars

(legend continued on next page)

have shown that age-dependent niche cell loss and reduced niche stem cell signaling factors lead to impaired stem cell maintenance, renewal, and differentiation in various stem cell niches, including murine spermatogonial stem cells (GSCs) (Dulken et al., 2019; Enwere et al., 2004; Ryu et al., 2006; Schüler et al., 2021; Silva-Vargas et al., 2016). Age-related niche deterioration ultimately causes stem cell loss (reviewed in Brunet et al., 2022). Currently, the molecular role of autophagy in adult stem cell niche cells remains unknown.

Several *Drosophila* studies highlight the importance of autophagy in development, cell death, nutrient stress, and aging (reviewed in McPhee and Baehrecke, 2009; Mulakkal et al., 2014). The female *Drosophila* GSC niche has been an excellent model for studying stem cell biology, stem cell-niche interaction, and GSC-niche aging (Ishibashi et al., 2020; Xie and Spradling, 2000). The female GSC niche is located at the anterior tip of the germarium, the anterior region of the ovariole that contains developing egg chambers. It comprises the terminal filament, the cap, and the escort cells that constitute the niche architecture (Xie, 2013). Cap cells, the primary niche cells, secrete BMP ligands decapentaplegic (Dpp) and glass bottom boat (Gbb), essential for GSC self-renewal and anchor GSCs through E-cadherin-based adherens junctions (Xie, 2013). The GSC niche typically contains six to eight cap cells and two to three GSCs. BMP-pMad signaling maintains GSCs in an undifferentiated state. Previous studies report reduced BMP/Dpp signaling and E-cadherin-mediated adhesion in aged germaria, leading to loss of GSCs and cap cell function (Pan et al., 2007). Niche deterioration, along with intrinsic stem cell aging, serves as an extrinsic driver of GSC aging (Boyle et al., 2007; Pan et al., 2007; Wallenfang et al., 2006).

In this study, we demonstrate the role of autophagy within the GSC niche. Our data show that Atg5 is required for basal autophagy in both GSCs and cap cells; it is essential for the survival of the cap cells but not GSCs. We found that a lack of autophagy in cap cells leads to the progressive reduction of their numbers, subsequently leading to GSC loss during aging. We further demonstrate that the lack of autophagy in the cap cells affects niche-GSC signaling required for GSC maintenance and leads to increased cap cell death.

RESULTS

Autophagy in GSCs is low and dispensable for GSC maintenance

We previously developed a germline-specific autophagy reporter, *nosP-mCherry-Atg8a*, expressing mCherry-tagged Atg8a under the *nanos* promoter (Nilangekar et al., 2019). Nutrient limitation strongly induces autophagy in the germarium (Barth et al., 2011; Hou et al., 2008; Nezis et al., 2009; Nilangekar et al., 2019). However, mCherry-Atg8a puncta in GSCs remain few and unchanged under starvation, or even pharmacological modulation of autophagy using rapamycin or chloroquine (CQ) compared to differentiating cysts in regions 2a and2b. (Nilangekar et al., 2019). We quantified autophagy flux in GSCs using *nosP-mCherry-Atg8a* flies, where mCherry-Atg8a marks autophagosomes and autolysosomes. After CQ treatment and cathepsin L immunostaining (which marks autolysosomes and lysosomes), the number of both autophagosomes and autolysosomes remained unchanged (Figure 1B). On average, one punctum per GSC was positive for both markers, indicating the presence of an autolysosome (Figures 1A and 1B). In contrast, CQ treatment increased the number of mCherry-Atg8a puncta in differentiating cells, suggesting increased autophagy (Nilangekar et al., 2019). These data confirm that basal autophagy and autophagy flux in GSCs are low.

Additionally, we assessed autophagy flux under genetically induced conditions, where overexpression of core autophagy genes such as *Atg1*, *Atg8a*, or *Atg5* has been shown to enhance autophagy activity (Bjedov et al., 2020; Pyo et al., 2013; Simonsen et al., 2008). Overexpression of Atg5 in the germ line using nosGal4VP16 > UASp-eGFP-Atg5 resulted in strong EGFP fluorescence in germline cells, including GSCs, confirming effective expression (Figure S1B). Further, we quantified autophagy flux in the presence or absence of CQ, in GSCs to determine whether Atg5 overexpression enhances autophagy. Despite Atg5 overexpression, autophagy flux in GSCs remained comparable to that of controls (Figures S1A and 1B).

As recommended by Klionsky et al. (2021), autophagy was evaluated using two independent markers (Klionsky et al., 2021). The autophagy receptor Ref(2)P (Refractory

represent the SD. Two independent experiments were performed. For the data presented, n = 19 control GSCs, which were heterozygous (+/−) or wild type (+/+) and n = 20 *Atg5* mutant GSCs. (F) Representative image showing mosaic GSC clones. *Atg5* null GSC (indicated as $Atg5^{-/-}$) with no visible RFP and neighboring RFP-positive heterozygous GSC (indicated as $Atg5^{+/-}$). Scale bar, 10 μm.
(G) Line graph showing the maintenance of *Atg5*-null GSCs compared to control GSC clones. Error bars represent SD. The data presented are aggregated from three independent biological replicates. n = 105 ± 6 (I), 80 ± 2 (II), and 70 ± 3 (III) germaria per genotype per time point for the three replicates (I, II, and III) (except III $Atg5^{5cc5}$ 2 days n = 54).
(H) Line graph showing the retention of *Atg5*-null GSCs under complete starvation compared to control GSC clones. The data presented are aggregated from two independent biological replicates. n = 105 ± 3 germaria per genotype per condition for each replicate (I and II) (except I control starved n = 92, II $Atg5^{5cc5}$ 3 days n = 87). ****$p < 0.0001$.

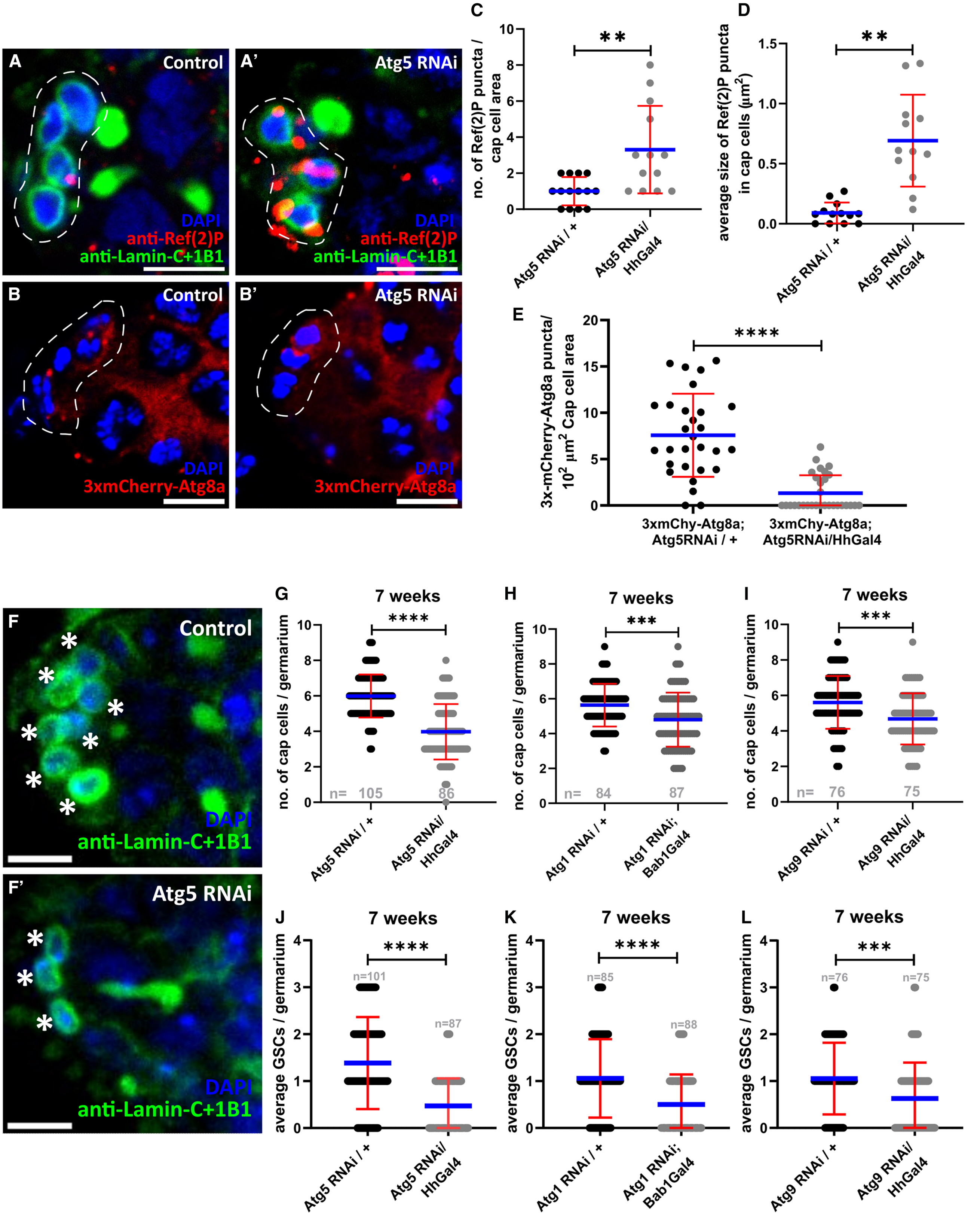

(legend on next page)

to Sigma P), a *Drosophila* homolog of p62/SQSTM1, was used to assess autophagic activity. Ref(2)P binds and delivers cargo to autophagosomes and is degraded along with it; hence its levels are inversely correlated with autophagy. Inhibition of autophagic degradation using agents such as CQ causes Ref(2)P accumulation, and its turnover in the presence or absence of such inhibitors serves as a reliable measure of autophagy flux (Klionsky et al., 2021). We used an anti-Ref(2)P antibody to detect Ref(2)P aggregates in germline cells from untreated and CQ-treated flies. Germaria from CQ-treated flies showed an increased number of Ref(2)P aggregates (Figure S1D); however, the number of Ref(2)P puncta within GSCs remained comparable between control and CQ-treated flies (Figure S1C). Furthermore, Atg5 overexpression did not alter the number of Ref (2)P puncta in GSCs, even after CQ treatment (Figure S1C). These results indicate that autophagy flux in GSCs is intrinsically low and is not enhanced by Atg5 overexpression.

We tested whether Atg5 is required for basal autophagy in GSCs by depleting *Atg5* mRNA in the germline using nosGal4VP16-driven Atg5IR expression (Nilangekar et al., 2019). Atg5 KD significantly increased the number and size of Ref(2)P puncta in GSCs (Figures S1E–S1G). The null allele $Atg5^{5cc5}$, which lacks over 85% of the coding region including the start site, confirmed the requirement of Atg5 for basal autophagy (Kim et al., 2016). Using the FLP-FRT system, we generated $Atg5^{-/-}$ GSC clones. Ref(2)P puncta were significantly increased in number and size in ($Atg5^{-/-}$) GSCs as compared to heterozygous ($Atg5^{-/+}$) or wild-type ($Atg5^{+/+}$) GSCs (Figures 1C–1E). In summary, These findings confirm that Atg5 is essential for Ref(2)P clearance, indicating that canonical autophagy operates in GSCs.

Our data show that Atg5 is required for basal autophagy in GSCs, consistent with reports linking autophagy to stem cell maintenance (Chen et al., 2018; García-Prat et al., 2016; Ho et al., 2017; B. V. Shravage and Turksen, 2023). We performed a GSC retention assay using *Atg5* null mutants, comparing the persistence of $Atg5^{-/-}$ GSC clones with control clones ($Atg5^{+/+}$) generated via the FLP-FRT system (*isoFRT19A*; $RFP^{-/-}$ isogenized wild-type chromosome containing *FRT19A*) (Figures 1F and 1G). Surprisingly, Atg5-deficient and control GSCs exhibited comparable retention for up to 19 days, indicating that Atg5-dependent autophagy is dispensable for GSC maintenance (Figure 1G). Atg5 is required for autophagy in GSCs but not for their maintenance, indicating that autophagy is dispensable for GSC maintenance.

Autophagy supports survival under nutrient stress (Klionsky et al., 2021). To assess its role, flies with control and *Atg5* mutant GSC clones were subjected to 4 days of complete starvation and compared with fed counterparts. Two-way ANOVA revealed comparable GSC numbers across fed and starved, control and mutant, and pre- and post-treatment groups (Figure 1H), indicating that starvation does not impair the maintenance of Atg5/autophagy-deficient GSCs.

Autophagy is required extrinsically in the cap cells for GSC maintenance

In the *Drosophila* male GSC niche, Demarco et al. reported that autophagy gene KD in CySCs significantly reduced GSC number (Sênos Demarco et al., 2020). We next aimed to establish and validate an "autophagy-defective niche" model. We targeted cap cells, the primary niche, using UAS-Gal4-driven RNAi to knock down Atg1, Atg13, Atg9, Atg5, Atg12, Atg16, and Atg8a, driven specifically by HhGal4 or Bab1Gal4. Autophagy disruption was verified by Ref(2)P aggregation, which accumulated in all Atg-RNAi cap cells except Atg12 and Atg13 KDs (Figures 2A–2D and S1H–S1K). Further, to assess autophagosome formation, we used the 3xmCherry-Atg8a reporter, which expresses mCherry-Atg8a under the endogenous *Atg8a* promoter. Atg5 KD cap cells showed markedly fewer mCherry-Atg8a puncta than controls (Figures 2B and 2E). Taken together, the autophagy-defective niche model was established and validated for Atg1, Atg9, Atg5, Atg16, and Atg8a.

Previous work established that cap cells are critical for GSC maintenance. We examined whether autophagy in

Figure 2. Autophagy in Cap cells is necessary for their and GSC maintenance

(A and A′) Representative image showing Ref(2)P in cap cells upon *Atg5* KD.

(B and B′) Representative image showing the autophagosomes in *Atg5 RNAi* cap cells. The region of cap cells is marked by dashed outlines. Scale bars, 5 μm. Interleaved scatterplots showing the number (C) and size (D) of Ref(2)P puncta in cap cells with *Atg5* KD. $n = 14 \pm 1$ cap cell planes/area per genotype.

(E) Interleaved scatterplot showing the number of 3xmCherry-Atg8a puncta (autophagosomes) in *Atg5* KD cap cells. $n = 30$ cap cell planes/area per genotype. Blue line represents the average, and error bars represent SD.

(F–L) (F and F′) Representative image showing the number of cap cells. Cap cells are marked by asterisks. Scale bars, 5 μm. Interleaved scatterplots showing the number of cap cells in old niches with *Atg5 RNAi* (G), *Atg1 RNAi* (H), and *Atg9 RNAi* (I) in the cap cells. Blue line represents the average, and error bars represent SD. Bar graphs showing the number of GSCs in old niches with *Atg5 RNAi* (J), *Atg1 RNAi* (K), and *Atg9 RNAi* (L) in the cap cells. Error bars represent SD. Sample sizes are the number of germaria as indicated in the graphs. $^{**}p < 0.01$, $^{***}p < 0.001$, $^{****}p < 0.0001$.

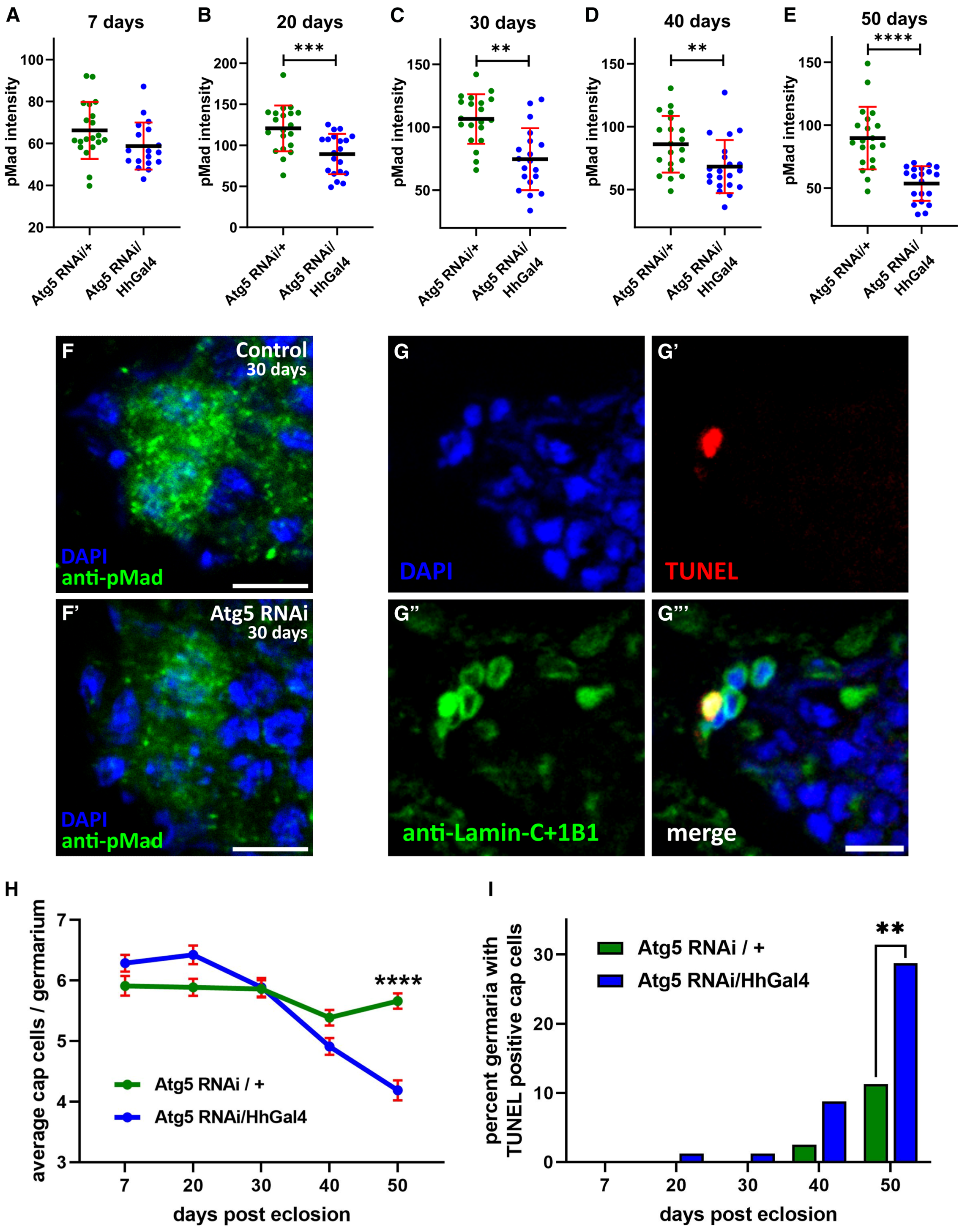

Figure 3. Lack of autophagy in cap cells affects their function and causes cell death during aging

(A–E) Interleaved scatterplots showing pMad intensity in GSCs of the mentioned genotypes at the five mentioned time points. Error bars represent SD. $n = 20 \pm 2$ GSCs per genotype per time point.

(F and F′) Representative image showing pMad in GSCs at 30 days. Scale bars, 5 μm.

(legend continued on next page)

cap cells is required for non-autonomous GSC maintenance. To test this, we used autophagy-defective niche models with Atg1, Atg9, or Atg5 KD and quantified GSC retention in 7-week-old germaria. Notably, GSCs were significantly reduced in these niches compared with age-matched controls (Figures 2J–2L). Hence, autophagy in cap cells is required for GSC maintenance during aging. Previous studies have shown that the number of GSCs correlates with the number of cap cells (≈1 GSC per 2.5 cap cells) (Ward et al., 2006; Xie and Spradling, 2000). As aged autophagy-defective niches contained fewer GSCs than the controls, we quantified the numbers of cap cells in these niches. Notably, autophagy-defective niches contained significantly fewer cap cells than age-matched controls (Figures 2F–2I). Because targeting multiple Atgs of different complexes yielded identical outcomes, the observed accelerated loss of GSC niche cells during aging is attributable to disrupted autophagy in cap cells. Thus, autophagy is required to sustain cap cells, which are essential for preserving GSCs.

As HhGal4 and Bab1Gal4 are active during development and gonad morphogenesis, we examined whether the observed effects occurred prior to the adult ovary stage. To assess this, cap cells and GSCs were quantified at 12 days post-eclosion, and in a separate, higher-temporal resolution experiment, as early as 7 days post-eclosion. The average cap cell and GSC numbers across experimental conditions were comparable to controls at early ages (Figures S2A, S2C, and S2D). These data indicate that the cap cell-to-GSC ratio was similar in controls and Atg5 KD niches, confirming that GSC-niche morphogenesis was unaffected during development. Thus, the accelerated loss of niche cells resulted from the absence of autophagy during aging.

Lack of autophagy affects cap cell function and causes cap cell death during aging

In female *Drosophila*, GSC self-renewal depends on secreted and membrane-bound cues. Cap cells secrete BMP ligands Dpp and Gbb, which drive GSC self-renewal and division. Loss of BMP signaling activates differentiation programs, causing GSC depletion (Xie and Spradling, 1998). Phosphorylated Mad (pMad), the key BMP/Dpp signaling effector, serves as a reliable marker of cap cell-GSC signaling strength. We quantified pMad levels in GSCs from autophagy-deficient niches across ages and observed a marked reduction from day 20 onward compared with controls (Figures 3A–3F). The same phenotype was reproduced using the cap cell-specific driver Bab1Gal4 (Figure S2E). Thus, autophagy impairment in cap cells disrupts BMP-pMad signaling in the GSC niche during mid to late aging.

In autophagy-defective niches, cap cells were lost rapidly with age (Figures 3H, S2A and S2C). We tested whether cap cell loss resulted from apoptosis using the TUNEL assay, which labels double-stranded DNA breaks characteristic of programmed cell death. TUNEL-positive cap cells were quantified in control and autophagy-defective niches across ages. Cap cell death was absent across all experimental conditions until 20 days. By 40 days, TUNEL-positive cap cells appeared in both control and autophagy-defective niches. At 50 days, however, Atg5 KD niches showed a significantly higher proportion of dying cap cells than age-matched controls (Figures 3G–3I and S2B). These results suggest that loss of autophagy triggers apoptosis in aged cap cells, indicating that autophagy acts as a survival factor during aging.

DISCUSSION

Our study reveals a differential role for autophagy in the *Drosophila* ovarian GSC niche. We show that autophagy is not required intrinsically in GSCs but is essential in niche cells, where it acts extrinsically to maintain GSCs. While Atg5 mediated Ref(2)P clearance in GSCs, Atg5-null GSCs were maintained despite complete loss of autophagy, even during starvation. *Atg5*-mediated autophagy was required for Ref(2)P clearance within the cap cells and, in contrast to its role in GSCs, was found to be critical for the survival of the cap cells. The autophagy-defective cap cells are compromised in function and thereby unable to maintain GSCs during aging. Disrupted autophagy drives GSC-niche decline, marked by cap cell apoptosis in old age and reduced niche-GSC BMP signaling from mid-life onward.

Atg5 is crucial for the self-renewal and differentiation of diverse adult stem cell types (Chen et al., 2018). Autophagy is widely recognized as essential for stem cell maintenance; thus, we expected it to be required for GSC maintenance. But surprisingly, in female *Drosophila* GSCs, autophagy is dispensable. Previously, we suggested that GSCs may be protected from starvation-induced autophagy (Nilangekar et al., 2019). This study used a comprehensive strategy to measure basal autophagy flux in GSCs. Autophagic vesicles were quantified across the full GSC volume, and both inhibition (CQ) and induction (Atg5 overexpression)

(G–G‴) Representative images showing a TUNEL-positive cap cell in the GSC niche. Scale bar, 5 μm.
(H) Line graph showing the number of cap cells across age. Error bars represent the SEM.
(I) Column graph showing percent germaria with TUNEL-positive cap cells across age in the mentioned genotypes ($^{**}p < 0.01$, Z score = −2.76). n = 80 germaria per genotype per time point for (H) and (I). $^{**}p < 0.01$, $^{***}p < 0.001$, $^{****}p < 0.0001$.

conditions were applied according to guidelines (Klionsky et al., 2021). Multiple markers, including nosP-mCherry-Atg8a, cathepsin-L, and Ref(2)P, enabled rigorous assessment (Klionsky et al., 2021). We demonstrated disruption of autophagy in *Atg5* mutant GSCs. Zhao et al. similarly showed that autophagy is low in GSCs and that their maintenance is unaffected in *Atg6* and *Fip200* mutants (Zhao et al., 2018). Demarco et al. also found that KD of multiple autophagy genes (*Atg1*, *Atg5*, *Atg6*, *Atg7*, and *Atg8a/8b*) in male GSCs did not alter their numbers at 10 days post-eclosion (Sênos Demarco et al., 2020). Thus, autophagy is dispensable in both male and female GSCs.

Nutrition shapes *Drosophila* oogenesis. Drummond-Barbosa and Spradling reported that GSC numbers are unaffected by diet, whereas their proliferation and differentiation into eggs are strongly influenced (Drummond-Barbosa and Spradling, 2001). Our analyses recapitulate that poor nutrition does not affect GSC numbers, even under conditions of complete starvation. Importantly, even Atg5/autophagy-deficient GSCs were resistant to GSC loss under severe nutrient stress, indicating their resilience, which may be crucial in the wild. This contrasts with the fact that starvation induces autophagy and autophagy-mediated cell death in the differentiated cells in the germarium (Barth et al., 2011; Hou et al., 2008; Nezis et al., 2009; Nilangekar et al., 2019). Taken together, this indicates differential regulation and strict repression of autophagy in the GSCs.

Autophagy in GSCs appears to be under tight control, since eGFP-Atg5 overexpression induced puncta in differentiating cells but not in GSCs (Figure S1B). eGFP-Atg5 puncta localize to phagophore assembly sites, isolation membranes, and immature autophagosomes; therefore, this observation also corroborates that autophagy in GSCs is low. Alternatively, this could be due to a lack of other core autophagy proteins required to form these intermediates. By contrast, murine Atg5 overexpression and neuronal Atg8a overexpression in *Drosophila* have each been shown to elevate autophagy (Pyo et al., 2013; Simonsen et al., 2008). Thus, autophagy in GSCs may be tightly and differently regulated compared to the differentiating germline cells.

The regulation of autophagy in GSCs is intriguing, as adjacent cysts exhibit moderate to high levels. Zhao and colleagues speculated that low autophagy in the GSCs may be explained by activation of BMP signaling (Zhao et al., 2018). Consistent with this view, Varga and colleagues demonstrated that BMP signaling suppresses autophagy in male *Drosophila* GSCs (Varga et al., 2022). As BMP-pMad signaling sustains female GSCs, our data imply that the self-renewal machinery actively inhibits autophagy in these cells. Elevated autophagy in cysts suggests that differentiation factors drive its upregulation, a view supported by Varga et al. (2022). Further, defective mitochondria in the female germ line undergo clearance via Atg1-dependent mitophagy (Ayachit and Shravage, 2023; Lieber et al., 2019; Palozzi et al., 2022). Insulin/InR-mTOR signaling, known to regulate GSC growth and division, may also control autophagy, either independently or via BMP signaling.

Although autophagy contributes to stem cell maintenance, a few studies demonstrate that *Atg5* deletion did not affect stem cell maintenance. In mice, *Atg12* and *Atg5* conditional KO HSCs persisted in normal numbers and Atg5 loss did not impair their maintenance (Ho et al., 2017). In case of murine neural stem cells, deletion of *Atg5*, *Atg16L1*, *Atg7*, or *FIP200* all showed defective autophagy and accumulation of mitochondria, but only *FIP200* deletion affected neural stem cell maintenance (Wang et al., 2016). In mice, Atg5 deletion did not compromise the maintenance of HSCs or neural stem cells, raising the yet unresolved question of why Atg5 is indispensable for autophagy in these cells but dispensable for their maintenance.

According to Demarco et al., autophagy supports male GSC maintenance non-autonomously via CySCs in the niche (Sênos Demarco et al., 2020). Similarly, in females, GSC maintenance depends on the niche, specifically the cap cells. The dichotomy between soma and germ line makes the ovarian tissue interesting. Autophagy is regulated differently in these cells and has distinct functions. For instance, Barth et al. demonstrated that Atg1 and Atg13 are required in follicle cells but not in germ cells for egg development (Barth et al., 2011). We observe a similar phenomenon in the GSC niche; autophagy is required in the somatic cells, i.e., cap cells, and is dispensable in GSCs. Another contrast between germ line and soma in the germarium arises from GSCs and follicle stem cells. Our findings, together with prior studies, indicate that autophagy is dispensable in GSCs, whereas follicle stem cells of somatic origin are strongly influenced by it. In these cells, autophagy increases with age and contributes to their loss (Singh et al., 2018). In summary, autophagy has distinct functions in the female *Drosophila* germ line and GSCs.

Cap cells are terminally differentiated, and Pan et al. reported their numbers remain largely stable throughout the fly lifespan (Pan et al., 2007). Significant cap cell loss does not occur until 7 weeks; even so, the loss is less than 20%. Autophagy supports the longevity of terminally differentiated post-mitotic cells such as neurons (Simonsen et al., 2008). In our study, despite autophagy disruption, cap cell death was only evident from 40 days onward, possibly due to incomplete Atg5 KD by RNAi, although severe Ref(2)P accumulation was observed. Apoptosis and autophagy both contribute to oogenesis

cell death in a cell type-, stage-, and context-dependent manner (Hou et al., 2008; Nezis et al., 2009; Pritchett et al., 2009). Autophagy acts upstream of apoptosis in early oogenesis (Nezis et al., 2009). Importantly, while cap cell death began at 40 days, functional deterioration was evident by 20 days, suggesting loss of homeostasis. We did not detect niche disruption during early life, possibly reflecting age-dependent physiological changes in *Drosophila* that emerge post-midlife (Aparicio et al., 2019; Rana et al., 2017). Alternate stress-mitigating pathways may also operate at earlier stages and in younger adults.

Although there is a significant and thorough understanding of the role of autophagy in stem cells as an intrinsic factor, there are very few reports connecting autophagy with the stem cell niche. Recent work demonstrated that sensory nerves activate autophagy in mesenchymal stem cells via fibroblast growth factor 1, a pathway critical for their maintenance (Pei et al., 2023). Emerging evidence, summarized in a recent review, points to systemic autophagy as a protective mechanism for vascular niche homeostasis (Dergilev et al., 2024).

Our finding that GSC maintenance is autophagy independent is baffling, especially considering the nature of these cells. The first level of distinction is that they are stem cells, and uniquely among the type of stem cells, they are GSCs. This is the first study that describes a novel role of autophagy in niche cells for stem cell maintenance. This study exemplifies a model to study the effect of autophagy-defective niches on the stem cells that they harbor. Atg5 is conserved in metazoans and plays an important role in autophagosome formation and closure. Thus, these findings could have critical implications for understanding the role of autophagy in niche-regulated stem cells, including in diseases such as cancer. Our findings add to the understanding of the fundamental role of autophagy in stem cell maintenance and where it has a non-cell-autonomous effect.

Limitations of the study

The influence of cap cells on GSC maintenance via autophagy likely involves pMad (BMP) signaling and cell survival, although the precise mechanism remains unresolved. Our findings suggest that loss of autophagy in cap cells disrupts homeostasis, which may lead to dysregulated transcription/translation and altered BMP ligand trafficking, resulting in reduced pMad levels. Similar effects may extend to adhesion factors such as E-cadherin. Beyond BMP, multiple pathways—insulin, Jak-Stat, Notch, and Hedgehog—also function in cap cells to support GSCs (Xie, 2013), raising the need to assess their regulation under autophagy loss. Variability in autophagy-dependent phenotypes may stem from incomplete Atg5 KD due to the UAS-Gal4 system, which could be addressed by mosaic analysis, although generating clones in adult cap cells is technically challenging. Alternatively, Atg mutant clones could be induced during niche specification. Another caveat lies in Gal4 drivers: Bab1-Gal4 and Hh-Gal4 mark the niche but are not cap cell specific, also targeting terminal filament cells. While these cells are important during niche formation, their role in the adult ovary is largely redundant, as cap cells provide key factors such as Piwi, Yb, and Dpp (Xie, 2013). Thus, cap cells remain the indispensable niche component, and taken together, our results indicate that autophagy-deficient niche cells compromise GSC maintenance primarily through cap cell dysfunction.

METHODS

Genotypes of all fly stocks

+; +; + (OregonR) |w^{1118}*; +; + |w; +; nosGal4VP16 (BL-4937) |w; nosP-mCherry-Atg8a; + |w; nosP-mCherry-Atg8a/CyO; nosGal 4VP16/TM6b, Hu |yw; +; UASp-eGFP-drAtg5 (derived from BL-59848) |ysc* v; Atg1 RNAi; + (BL-44034) |ysc* v; +; Atg5 RNAi (BL-34899) |ysc* v; Atg8a RNAi; + (BL-58309) |ysc* v; +; Atg9 RNAi (BL-34901) |ysc* v; Atg13 RNAi; + (BL-40861) |ysc* v; +; Atg16 RNAi (BL-34358) |w; If/CyO; HhGal4, UAS-GFP/TM6B, Tb |w; +; HhGal4, UAS-GFP/TM6B, Tb |w; +; Bab1Gal4/TM6B (BL-6803) |yw; 3xmCherry-Atg8a; + |w; 3xmCherry-Atg8a/CyO; HhGal4, UAS-GFP/TM6B |y w*$^{+}$ *Atg5*^{5cc5} *FRT19A/FM7i; +; + |y w iso FRT19A; +; + |w hsFLP12, Ubi-RFP FRT19A; +; + (BL-31418) |y w hsFLP12, His2Av.GFP FRT19A/FM7a; +; + (BL-32045) |*.

Aging

Large crosses were set to collect a large number of age-synchronized progeny of the desired genotype, which were collected within 24–48 h of eclosion. Until dissection the flies were housed under standard conditions as 15 females and 7–10 males in a vial, which were flipped every 3 to 4 days to fresh food vials supplemented with dry yeast pellets. The vials were kept horizontal throughout the duration in order to avoid death of flies due to sticking onto the food surface. Subsets of the collected and aged flies were dissected at each of the time points.

Immunostaining and TUNEL staining

The immunostaining procedure was followed as published previously (Nilangekar et al., 2019). *In Situ* Cell Death Detection Kit, TMR red (Roche, 12156792910) was used for TUNEL staining with a modified procedure. The details of immunostaining and TUNEL staining are elaborated in the supplementary text.

Confocal microscopy

The imaging was performed on the Leica SP8 confocal microscope or the confocal mode of the Zeiss LSM900

Airyscan microscope. Detailed settings and parameters are elaborated in the supplementary text.

Statistical analysis

For all comparative analyses, Student's *t* test assuming unequal variance was used. To statistically compare the effect of fed versus starved between control versus *Atg5* mutant GSC clones, two-way ANOVA was used. For statistical comparison of the proportion of TUNEL positive cap cells out of total cap cells among different genotypes, *Z* test for two proportions was used. Microsoft Excel was used to store, arrange, and analyze data. GraphPad Prism was used for plotting all the graphs.

RESOURCE AVAILABILITY

Lead contact

Further information and requests for resources should be directed to and will be fulfilled by the lead contact, Bhupendra V Shravage (bvshravage@aripune.org, bhupendra.shravage@gmail.com).

Materials availability

No new materials or reagents were generated in the study.

Data and code availability

All datasets supporting the findings of this study are available from the corresponding author upon reasonable request.

ACKNOWLEDGMENTS

We thank Dr. Gábor Juhász for providing the *Atg5* mutant and 3xmCherry-Atg8a fly stocks, Dr. Manish Jaiswal for providing the FRT19A stocks, and Dr. Richa Rikhy and the IISER-Pune fly facility for providing many essential fly stocks. We thank Agharkar Research Institute, Pune, India, and the Developmental Biology fraternity for support and confocal facility access. This work was supported by DST-SERB grant number ECR/2015/000239, BT/RLF/Re-entry/58/2013, and BT/PR12718/MED/31/298/2015 to B.V.S. K.S.N. was supported by ICMR-SRF 2020-6879/CMB-BMS and was a registered Ph.D. student affiliated with the Department of Biotechnology, Savitribai Phule Pune University, Pune, India (registration no. 175, PGS/4204). B.V.S. is affiliated to Savitribai Phule Pune University, Pune, India, and is recognized by SPPU as a PhD guide in biotechnology and zoology.

AUTHOR CONTRIBUTIONS

K.S.N. and B.V.S. conceived the project and designed the experiments. K.S.N. conducted all experiments and analysis. K.S.N. and B.V.S. wrote the manuscript.

DECLARATION OF INTERESTS

The authors declare no conflicts of interest.

SUPPLEMENTAL INFORMATION

Supplemental information can be found online at https://doi.org/10.1016/j.stemcr.2025.102712.

Received: August 20, 2025
Revised: October 14, 2025
Accepted: October 15, 2025
Published: November 13, 2025

REFERENCES

Adelipour, M., Saleth, L.R., Ghavami, S., Alagarsamy, K.N., Dhingra, S., and Allameh, A. (2022). The role of autophagy in the metabolism and differentiation of stem cells. Biochim. Biophys. Acta. Mol. Basis Dis. *1868*, 166412. https://doi.org/10.1016/J.BBADIS.2022.166412.

Aparicio, R., Rana, A., and Walker, D.W. (2019). Upregulation of the Autophagy Adaptor p62/SQSTM1 Prolongs Health and Lifespan in Middle-Aged Drosophila. Cell Rep. *28*, 1029–1040.e5. https://doi.org/10.1016/J.CELREP.2019.06.070.

Ayachit, M.S., and Shravage, B.V. (2023). Atg1 modulates mitochondrial dynamics to promote germline stem cell maintenance in Drosophila. Biochem. Biophys. Res. Commun. *643*, 192–202. https://doi.org/10.1016/J.BBRC.2022.11.076.

Barth, J.M.I., Szabad, J., Hafen, E., and Köhler, K. (2011). Autophagy in Drosophila ovaries is induced by starvation and is required for oogenesis. Cell Death Differ. *18*, 915–924. https://doi.org/10.1038/CDD.2010.157.

Bjedov, I., Cochemé, H.M., Foley, A., Wieser, D., Woodling, N.S., Castillo-Quan, J.I., Norvaisas, P., Lujan, C., Regan, J., Toivonen, J. M., et al. (2020). Fine-tuning autophagy maximises lifespan and is associated with changes in mitochondrial gene expression in Drosophila. PLoS Genet. *16*, e1009083. https://doi.org/10.1371/JOURNAL.PGEN.1009083.

Boyle, M., Wong, C., Rocha, M., and Jones, D.L. (2007). Decline in self-renewal factors contributes to aging of the stem cell niche in the Drosophila testis. Cell Stem Cell *1*, 470–478. https://doi.org/10.1016/J.STEM.2007.08.002.

Brunet, A., Goodell, M.A., and Rando, T.A. (2022). Ageing and rejuvenation of tissue stem cells and their niches. Nat. Rev. Mol. Cell Biol. *24*, 45–62. https://doi.org/10.1038/s41580-022-00510-w.

Chang, T.K., Shravage, B.V., Hayes, S.D., Powers, C.M., Simin, R.T., Wade Harper, J., and Baehrecke, E.H. (2013). Uba1 functions in Atg7- and Atg3-independent autophagy. Nat. Cell Biol. *15*, 1067–1078. https://doi.org/10.1038/NCB2804.

Chen, X., He, Y., and Lu, F. (2018). Autophagy in Stem Cell Biology: A Perspective on Stem Cell Self-Renewal and Differentiation. Stem Cells Int. *2018*, 9131397. https://doi.org/10.1155/2018/9131397.

Dergilev, K., Gureenkov, A., and Parfyonova, Y. (2024). Autophagy as a Guardian of Vascular Niche Homeostasis. Int. J. Mol. Sci. *25*, 10097. https://doi.org/10.3390/IJMS251810097.

Drummond-Barbosa, D., and Spradling, A.C. (2001). Stem Cells and Their Progeny Respond to Nutritional Changes during Drosophila Oogenesis. Dev. Biol. *231*, 265–278. https://doi.org/10.1006/dbio.2000.0135.

Dulken, B.W., Buckley, M.T., Navarro Negredo, P., Saligrama, N., Cayrol, R., Leeman, D.S., George, B.M., Boutet, S.C., Hebestreit, K., Pluvinage, J.V., et al. (2019). Single-cell analysis reveals T cell

infiltration in old neurogenic niches. Nature *571*, 205–210. https://doi.org/10.1038/s41586-019-1362-5.

Enwere, E., Shingo, T., Gregg, C., Fujikawa, H., Ohta, S., and Weiss, S. (2004). Aging Results in Reduced Epidermal Growth Factor Receptor Signaling, Diminished Olfactory Neurogenesis, and Deficits in Fine Olfactory Discrimination. J. Neurosci. *24*, 8354–8365. https://doi.org/10.1523/JNEUROSCI.2751-04.2004.

García-Prat, L., Martínez-Vicente, M., Perdiguero, E., Ortet, L., Rodríguez-Ubreva, J., Rebollo, E., Ruiz-Bonilla, V., Gutarra, S., Ballestar, E., Serrano, A.L., et al. (2016). Autophagy maintains stemness by preventing senescence. Nature *529*, 37–42. https://doi.org/10.1038/nature16187.

Ho, T.T., Warr, M.R., Adelman, E.R., Lansinger, O.M., Flach, J., Verovskaya, E.V., Figueroa, M.E., and Passegué, E. (2017). Autophagy maintains the metabolism and function of young and old stem cells. Nature *543*, 205–210. https://doi.org/10.1038/nature21388.

Hou, Y.C.C., Chittaranjan, S., Barbosa, S.G., McCall, K., and Gorski, S.M. (2008). Effector caspase Dcp-1 and IAP protein Bruce regulate starvation-induced autophagy during Drosophila melanogaster oogenesis. J. Cell Biol. *182*, 1127–1139. https://doi.org/10.1083/JCB.200712091.

Huang, Q., Liu, Y., Zhang, S., Yap, Y.T., Li, W., Zhang, D., Gardner, A., Zhang, L., Song, S., Hess, R.A., and Zhang, Z. (2021). Autophagy core protein ATG5 is required for elongating spermatid development, sperm individualization and normal fertility in male mice. Autophagy *17*, 1753–1767. https://doi.org/10.1080/15548627.2020.1783822.

Ishibashi, J.R., Taslim, T.H., and Ruohola-Baker, H. (2020). Germline stem cell aging in the Drosophila ovary. Curr. Opin. Insect Sci. *37*, 57–62. https://doi.org/10.1016/J.COIS.2019.11.003.

Kim, M., Sandford, E., Gatica, D., Qiu, Y., Liu, X., Zheng, Y., Schulman, B.A., Xu, J., Semple, I., Ro, S.H., et al. (2016). Mutation in ATG5 reduces autophagy and leads to ataxia with developmental delay. eLife *5*, e12245. https://doi.org/10.7554/ELIFE.12245.001.

Klionsky, D.J., Abdel-Aziz, A.K., Abdelfatah, S., Abdellatif, M., Abdoli, A., Abel, S., Abeliovich, H., Abildgaard, M.H., Abudu, Y.P., Acevedo-Arozena, A., et al. (2021). Guidelines for the use and interpretation of assays for monitoring autophagy (4th edition)1. Autophagy *17*, 1–382. https://doi.org/10.1080/15548627.2020.1797280.

Lieber, T., Jeedigunta, S.P., Palozzi, J.M., Lehmann, R., and Hurd, T. R. (2019). Mitochondrial fragmentation drives selective removal of deleterious mtDNA in the germline. Nature *570*, 380–384. https://doi.org/10.1038/S41586-019-1213-4.

McPhee, C.K., and Baehrecke, E.H. (2009). Autophagy in Drosophila melanogaster. Biochim. Biophys. Acta *1793*, 1452–1460. https://doi.org/10.1016/J.BBAMCR.2009.02.009.

Mulakkal, N.C., Nagy, P., Takats, S., Tusco, R., Juhász, G., and Nezis, I.P. (2014). Autophagy in drosophila: From historical studies to current knowledge. BioMed Res. Int. *2014*, 273473. https://doi.org/10.1155/2014/273473.

Nezis, I.P., Lamark, T., Velentzas, A.D., Rusten, T.E., Bjørkøy, G., Johansen, T., Papassideri, I.S., Stravopodis, D.J., Margaritis, L.H., Stenmark, H., and Brech, A. (2009). Cell death during Drosophila melanogaster early oogenesis is mediated through autophagy. Autophagy *5*, 298–302. https://doi.org/10.4161/auto.5.3.7454.

Nilangekar, K., Murmu, N., Sahu, G., and Shravage, B.V. (2019). Generation and characterization of germline-specific autophagy and mitochondrial reactive oxygen species reporters in Drosophila. Front. Cell Dev. Biol. *7*, 47. https://doi.org/10.3389/FCELL.2019.00047.

Palozzi, J.M., Jeedigunta, S.P., Minenkova, A.V., Monteiro, V.L., Thompson, Z.S., Lieber, T., and Hurd, T.R. (2022). Mitochondrial DNA quality control in the female germline requires a unique programmed mitophagy. Cell Metab. *34*, 1809–1823.e6. https://doi.org/10.1016/j.cmet.2022.10.005.

Pan, L., Chen, S., Weng, C., Call, G., Zhu, D., Tang, H., Zhang, N., and Xie, T. (2007). Stem cell aging is controlled both intrinsically and extrinsically in the Drosophila ovary. Cell Stem Cell *1*, 458–469. https://doi.org/10.1016/J.STEM.2007.09.010.

Pei, F., Ma, L., Jing, J., Feng, J., Yuan, Y., Guo, T., Han, X., Ho, T.V., Lei, J., He, J., et al. (2023). Sensory nerve niche regulates mesenchymal stem cell homeostasis via FGF/mTOR/autophagy axis. Nat. Commun. *14*, 344. https://doi.org/10.1038/s41467-023-35977-4.

Pritchett, T.L., Tanner, E.A., and McCall, K. (2009). Cracking open cell death in the Drosophila ovary. Apoptosis *14*, 969–979. https://doi.org/10.1007/S10495-009-0369-Z.

Pyo, J.O., Yoo, S.M., Ahn, H.H., Nah, J., Hong, S.H., Kam, T.I., Jung, S., and Jung, Y.K. (2013). Overexpression of Atg5 in mice activates autophagy and extends lifespan. Nat. Commun. *4*, 2300. https://doi.org/10.1038/ncomms3300.

Rana, A., Oliveira, M.P., Khamoui, A.V., Aparicio, R., Rera, M., Rossiter, H.B., and Walker, D.W. (2017). Promoting Drp1-mediated mitochondrial fission in midlife prolongs healthy lifespan of Drosophila melanogaster. Nat. Commun. *8*, 448. https://doi.org/10.1038/s41467-017-00525-4.

Rusten, T.E., Lindmo, K., Juhász, G., Sass, M., Seglen, P.O., Brech, A., and Stenmark, H. (2004). Programmed Autophagy in the Drosophila Fat Body Is Induced by Ecdysone through Regulation of the PI3K Pathway. Dev. Cell *7*, 179–192. https://doi.org/10.1016/J.DEVCEL.2004.07.005.

Ryu, B.-Y., Orwig, K.E., Oatley, J.M., Avarbock, M.R., and Brinster, R.L. (2006). Effects of Aging and Niche Microenvironment on Spermatogonial Stem Cell Self-Renewal. Stem Cells *24*, 1505–1511. https://doi.org/10.1634/STEMCELLS.2005-0580.

Schüler, S.C., Kirkpatrick, J.M., Schmidt, M., Santinha, D., Koch, P., Di Sanzo, S., Cirri, E., Hemberg, M., Ori, A., and von Maltzahn, J. (2021). Extensive remodeling of the extracellular matrix during aging contributes to age-dependent impairments of muscle stem cell functionality. Cell Rep. *35*, 109223. https://doi.org/10.1016/J.CELREP.2021.109223.

Scott, R.C., Schuldiner, O., and Neufeld, T.P. (2004). Role and regulation of starvation-induced autophagy in the Drosophila fat body. Dev. Cell *7*, 167–178. https://doi.org/10.1016/J.DEVCEL.2004.07.009.

Sênos Demarco, R., Uyemura, B.S., and Jones, D.L. (2020). EGFR Signaling Stimulates Autophagy to Regulate Stem Cell Maintenance

and Lipid Homeostasis in the Drosophila Testis. Cell Rep. *30*, 1101–1116.e5. https://doi.org/10.1016/J.CELREP.2019.12.086.

Shravage, B.V., and Turksen, K. (2023). In Autophagy in stem cell maintenance and differentiation, 1st ed., B. Shravage and K. Turksen, eds. (Springer).

Silva-Vargas, V., Maldonado-Soto, A.R., Mizrak, D., Codega, P., and Doetsch, F. (2016). Age-Dependent Niche Signals from the Choroid Plexus Regulate Adult Neural Stem Cells. Cell Stem Cell *19*, 643–652. https://doi.org/10.1016/J.STEM.2016.06.013.

Simonsen, A., Cumming, R.C., Brech, A., Isakson, P., Schubert, D. R., and Finley, K.D. (2008). Promoting basal levels of autophagy in the nervous system enhances longevity and oxidant resistance in adult Drosophila. Autophagy *4*, 176–184. https://doi.org/10.4161/AUTO.5269.

Singh, T., Lee, E.H., Hartman, T.R., Ruiz-Whalen, D.M., and O'Reilly, A.M. (2018). Opposing Action of Hedgehog and Insulin Signaling Balances Proliferation and Autophagy to Determine Follicle Stem Cell Lifespan. Dev. Cell *46*, 720–734.e6. https://doi.org/10.1016/J.DEVCEL.2018.08.008.

Varga, V.B., Schuller, D., Szikszai, F., Szinyákovics, J., Puska, G., Vellai, T., and Kovács, T. (2022). Autophagy is required for spermatogonial differentiation in the Drosophila testis. Biol. Futur. *73*, 187–204. https://doi.org/10.1007/S42977-022-00122-7.

Wallenfang, M.R., Nayak, R., and DiNardo, S. (2006). Dynamics of the male germline stem cell population during aging of Drosophila melanogaster. Aging Cell *5*, 297–304. https://doi.org/10.1111/J.1474-9726.2006.00221.X.

Wang, C., Chen, S., Yeo, S., Karsli-Uzunbas, G., White, E., Mizushima, N., Virgin, H.W., and Guan, J.L. (2016). Elevated p62/SQSTM1 determines the fate of autophagy-deficient neural stem cells by increasing superoxide. J. Cell Biol. *212*, 545–560. https://doi.org/10.1083/JCB.201507023.

Ward, E.J., Shcherbata, H.R., Reynolds, S.H., Fischer, K.A., Hatfield, S.D., and Ruohola-Baker, H. (2006). Stem Cells Signal to the Niche through the Notch Pathway in the Drosophila Ovary. Curr. Biol. *16*, 2352–2358. https://doi.org/10.1016/J.CUB.2006.10.022.

Xie, T., and Spradling, A.C. (2000). A niche maintaining germ line stem cells in the Drosophila ovary. Science *290*, 328–330. https://doi.org/10.1126/SCIENCE.290.5490.328.

Xie, T. (2013). Control of germline stem cell self-renewal and differentiation in the Drosophila ovary: concerted actions of niche signals and intrinsic factors. Wiley Interdiscip. Rev. Dev. Biol. *2*, 261–273. https://doi.org/10.1002/WDEV.60.

Xie, T., and Spradling, A.C. (1998). decapentaplegic is essential for the maintenance and division of germline stem cells in the Drosophila ovary. Cell *94*, 251–260. https://doi.org/10.1016/S0092-8674(00)81424-5.

Zhao, S., Fortier, T.M., and Baehrecke, E.H. (2018). Autophagy Promotes Tumor-like Stem Cell Niche Occupancy. Curr. Biol. *28*, 3056–3064.e3. https://doi.org/10.1016/J.CUB.2018.07.075.

Stem Cell Reports, Volume *20*

Supplemental Information

Autophagy is dispensable in germline stem cells but is required in the cap cells for their maintenance in the *Drosophila* ovarian niche

Kiran Suhas Nilangekar and Bhupendra V. Shravage

Supplementary text

Materials and methods

Fly maintenance

Flies were maintained at standard conditions; 25℃, 60-70% relative humidity, 12-12-hour light-dark cycle. Fly food composition per liter of food; 80g sugar, 75g corn flour, 30g yeast, 30g malt extract, 10g agar, 0.12% methyl benzoate, 0.4% propionic acid and 0.08% orthophosphoric acid. For setting crosses, 0–5-day old males and virgin females were used. Until dissection, the flies of desired genotypes were housed as 15 females and 7-10 males in a vial which were flipped every two to three days to fresh food vials supplemented with dry yeast pellets. Two days prior to dissection, the ovaries were fattened by transferring the flies, each day on fresh food vials supplemented with dry yeast pellets. Diethyl ether vapors were used to anesthetize flies. For chloroquine treatment, a stock solution of 50 mg/ml chloroquine in water was prepared from chloroquine diphosphate salt (Sigma Aldrich, C6628) for experiments in Figure 1A and Figure S1A. The 50 mg/ml chloroquine stock solution was added to fly food to a final concentration of 3 mg/ml when the food cooled down to 50-60℃ during preparation. Flies from treated and untreated sets were first fed on food supplemented with yeast pellets for two days before being subjected to treatment. The flies for chloroquine treatment were transferred to fresh chloroquine containing food vials for two days while the untreated flies were transferred to fresh vials containing only normal fly food. For experiments in Figure S1C and D, a stock solution close to 50 mg/ml was prepared from chloroquine tablets (Chloroquine phosphate IP 500 mg, Lariago®-DS, Ipca Laboratories ltd.), the tablets were dissolved in water and the insoluble excipients were filtered through 0.22 µm filter. For these experiments, freshly eclosed flies were fed on food supplemented with yeast pellets for three days prior treatment and subsequently transferred to fresh vials having either chloroquine containing food (3 mg/ml) or only normal fly food, for three days.

Recombination of Atg5 mutant and FRT19A and its validation

The *Atg5*^{5cc5} mutant was first crossed to *isoFRT19A* and the virgin female progeny was crossed to *FM7i* males. After a day of setting the cross, the flies were transferred to vials containing 500 µg/ml geneticin (G418) (Gibco, 11811-023) for egg laying. G418 was used to screen the FRT19A positive recombinants since the FRT19A cassette has neomycin as a selection marker. All the surviving virgin female progeny developed from the G418 vials were crossed to *FM7i* males in multiple single pair crosses. Males from the progeny of each of the single pair crosses were used to perform single fly PCR to confirm the presence of mutation using primers that specifically bind in the region of *Atg5* which was

deleted in *Atg5*^{5cc5} (forward primer: 5'GCACTACATGTCCTGCCTGA, reverse primer: 5'AGATTCGCAGGGGAATGTTT).

FLP-FRT based GSC clone generation and GSC retention assay

Large cross of *Atg5*^{5cc5} *FRT19A* with *whsFLP, Ubi-RFP FRT19A* for Atg5 mutant GSC clones and another cross of *isoFRT19A* with *whsFLP, Ubi-RFP FRT19A* for control GSC clones were set in multiple vials. The crosses were transferred to a set of fresh vials supplemented with yeast paste and removed after six-seven hours to obtain synchronized egg lay. The vials were subjected to heat shock for four consecutive days on seventh through tenth day after egg lay i.e., during pupal development. Heat shocks were applied for 50 minutes twice a day, six-eight hours apart, at 37℃ in a water bath. Only the flies which eclosed after the complete heat shock regime were collected. Therefore, the days-post-eclosion is the same as days-post-heat-shock. Females of the appropriate genotype were collected and aged until the stipulated timepoints. For each time-point, 8-10 of the collected females were dissected two, nine and nineteen days-post-heat-shock and immunostained for hts (1B1) and Lamin C for identification and quantification of RFP^+ and RFP^- GSCs.

For fed/starved treatment in the GSC clone retention assay, 'complete starvation vials' containing 0.8% agar were prepared; 0.8% agar (w/v) was dissolved in water by heating, and methyl benzoate to a final concentration of 0.12% was added when the solution cooled to 50-60℃. Flies from all sets for this experiment were initially fed for three consecutive days post-eclosion by transferring them to fresh vials containing food supplemented with yeast pellets. At this stage, a subset of control and *Atg5* mutant flies were dissected to quantify the GSC clones' frequency before the fed/starved treatment. Consequently, sets of flies were transferred to either complete starvation vials or vials with food supplemented with yeast pellets for four consecutive days for the fed/starved treatment and then dissected. All sets were immunostained for hts (1B1) and Lamin C for identification and quantification of RFP^+ and RFP^- GSCs.

Immunostaining of Ref(2)P in *Atg5* mutant GSCs was performed in two experiments. First using *Ubi-RFP FRT19A* where the progeny was heat shocked in the adult stage ten days post eclosion for three consecutive days in the regime stated above, and dissections were performed two days after the last heat shock. Second where the immunostaining was performed two-days post eclosion after pupal heat shock. The same procedure was repeated while using *His2AvGFP FRT19A* and the phenotype could be replicated (data not shown).

Immunostaining

Flies were briefly anesthetized and dissected in Grace's medium (Gibco, 11667-037). Ovaries were extracted and non-vitellogenic region of the ovarioles were partially teased apart using minutien pins. The ovaries were then transferred to 0.5 ml tubes with the aid of a cut-tip passivated with bovine serum albumin (BSA) (MP Biomedicals, 199897). The ovaries were fixed with 350 µl 4% paraformaldehyde (Sigma Aldrich, P6148) in 1xPBS (phosphate-buffered saline) pH 7.4 for 15 minutes at room temperature with the gentle nutation. All the following steps are performed with gentle nutation 15-20 RPM. The fixative was washed off with three washes of 400 µl 0.1% PBTx (0.1% triton-X-100{SRL, 64518} in 1xPBS) for five minutes each. Blocking was performed in 300 µl of 0.5% BSA in 1% PBTx for one hour at room temperature. The sample was incubated with at least 100 µl of the appropriate primary antibody solution in which the antibody was diluted in 0.3% PBTx containing 0.5% BSA. The incubation with primary antibody solution was performed at 4℃ overnight with gentle nutation 5 RPM. The primary antibody was washed off with 400 µl 0.1% PBTx for 15 minutes at room temperature. Following which the samples were again blocked for secondary antibody staining with 400 µl of 10% normal goat serum (NGS) (MP Biomedicals, 2939149) in 0.1% PBTx for two hours at room temperature. The secondary antibody diluted in the 10% NGS solution in 0.1% PBTx was incubated with the samples for two hours at room temperature. Three washes of 15 minutes each with 400 µl of 0.1% PBTx were performed at room temperature. For DAPI staining, the samples were incubated in 1 µg/ml DAPI in 0.1% PBTx solution for 10 minutes at room temperature and consequently washed off twice for five minutes each with 400 µl 0.1% PBTx. The samples were stored at 4℃ until mounting. All the PBTx solution was removed carefully and mounting medium SlowFade Glass mountant (Invitrogen, S36917) was added. All the ovaries were transferred onto a slide along with the mounting medium. In order to obtain flat mounting of the germarium, the region of the ovarioles which have the string of pre-vitellogenic stages and germarium at the tip were separated using minutien pins and the remaining large part of the ovaries was picked and removed from the slide. For optimal Confocal microscopy, ~170 µm thick no.1 coverslips were used. Nail varnish was used to seal the slide. The slides were stored at 4°C protected from light until microscopy. All the anti-pMad staining were executed with a same workflow for consistency across timepoints.

The following primary antibodies were used with the mentioned dilutions; anti-Cathepsin L (Abcam, ab58991; 1:400), anti-Ref(2)P (Abcam, ab178440; 1:1000), anti-Hts (DSHB, 1B1; 1:50), anti-Lamin C (DSHB, LC28.26; 1:50), anti-pMad (Abcam, ab52903; 1:50). The following secondary antibodies were used at 1:250 dilution; Goat anti-Rabbit Alexa Fluor 555 (Thermo Fisher Scientific, A21429), Goat anti-Rabbit Alexa Fluor 647 (Thermo Fisher Scientific, A21245) and Goat anti-Mouse Alexa Fluor 647 (Thermo Fisher Scientific, A21236).

TUNEL staining

In Situ Cell Death Detection Kit, TMR red (Roche, 12156792910) was used for TUNEL staining. The procedure was repeated exactly the same for all five time points. Ovaries were dissected and teased as described for immunostaining. All the following steps were performed with gentle nutation and the volumes indicated are for each sample. Fixation: 15 minutes at room temperature, 300 µl 4% paraformaldehyde in PBS pH 7.4. Followed by three washes of 5 minutes each at room temperature with 300 µl 0.1% PBTx for each. Subsequently, blocking and permeabilization: 300 µl of 1% PBTx containing 0.5% BSA, for one hour at room temperature. Followed by one wash with 400 µl 1x PBS for 2 minutes at room temperature. Total of 250 µl TUNEL reaction solution was prepared during each experiment, which comprised of 225 µl TUNEL TMR label solution and 25 µlTdT enzyme solution. 50 µl TUNEL reaction solution was used per sample. TUNEL reaction was performed by adding the TUNEL reaction solution to the sample and incubating for 2 hours at 37°C in a shaking incubator, 80 RPM and protected from light. All following steps were performed with samples protected from light. The samples were proceeded for immunostaining after blocking in 300 µl of 1% PBTx containing 0.5% BSA for 30 minutes at room temperature.

Confocal microscopy

Settings were identical across control versus test, a set of experiments or a session of imaging. All the microscopy performed on the Leica SP8 was done using 63x oil immersion objective NA=1.4. For all intensity and puncta count analyses performed on Leica SP8, the settings were as follows; resolution=1024x1024 pixels, scan speed=100 Hz, bit depth=8-bit. For obtaining z-stacks for GSC or cap cell counting; resolution=512x512 pixels, scan speed=400-600 Hz, bit depth=8-bit, z-step size=0.5 µm.

For the measurement of autophagy flux in GSCs, z-stacks spanning entire GSCs were acquired by visual confirmation from the DAPI (nucleus) channel. The z-step size was set to 0.4 µm which ensured that puncta were not missed out between steps. 63x oil immersion objective NA=1.4, 512x512 pixels at 0.05 µm per pixel resolution, scan speed=8, bit depth=16-bit.

For all GSC and cap cell counts, z-stacks of the tip of the germarium which covered the entire GSC niche were acquired by visual confirmation (z-step size=0.5 µm). While imaging for Ref(2)P puncta in the cap cells, the central plane of the cap cells was focused using the Lamin C channel. While imaging 3xmCherry-Atg8a in cap cells, the plane maximally covering the cluster of cap cells centrally was identified in the DAPI channel. The GSCs were identified by their location and size of the nucleus and the optical section across the central plane was acquired by focusing in the DAPI channel for the following

experiments; Ref(2)P in *Atg5* knockdown, *Atg5* overexpression, *Atg5* mutant, and pMad. Imaging settings for all puncta count experiments were exactly the same for all experimental conditions and the imaging of a set was completed in one imaging session. Imaging settings for all pMad intensity measurements were identical, except for 7-day timepoints where the frame accumulation was set to one, and the same was set to two for all others. The image acquisition was performed in the dynamic range where the non-tissue and tissue background were comparable among images of a set.

Image analysis

All the image analyses involving intensity measurement or puncta count were performed in ImageJ and Fiji. All steps performed for image analyses were exactly the same for all the control and test images in a data set. For GSC counts, *Atg5* mutant and control GSC clone counts, cap cell counts and TUNEL positive cap cell counts, LAS-X and Zeiss Zen 3.7 software were used to visualize and manually count the cells. GSCs were unequivocally identified by the following characteristics; their location, size of the nucleus, presence and orientation of the spectrosome. Cap cells were identified by their nuclear shape, location and Lamin C ring.

For autophagic flux determination in the GSCs, nosP-mCherry-Atg8a and Cathepsin L puncta within the GSCs were manually marked in the plane with maximal area and intensity. Additionally, colocalized mCherry-Atg8a and Cathepsin L puncta were also manually identified. Similarly, 3xmCherry-Atg8a puncta in the cap cells were manually quantified. For quantification of Ref(2)P puncta in the germaria, GSCs or cap cells; ROI was drawn to define either the germaria, GSCs or cap cells in the DAPI channel and the ROI was imported into the 'ROI manager'. The Ref(2)P channel was first subjected to the 'Subtract background' function with rolling ball radius 50 pixels or 35 pixels according to the optimal value for the data set. The resultant images were thresholded referring to the display under 'Max entropy' algorithm and then manually adjusted for each image such that the background was excluded but the true signal of the puncta did not erode. The thresholded images were then used for the function 'Analyze particles' in which the following parameters were set; Size (micron^2): 0.02-infinity or 0.03-infinity across a particular set according to the optimal value, Circularity: 0-1, from which the output was obtained as number and size of puncta within the marked ROI. pMad intensity in the GSCs was quantified in ImageJ by marking ROIs and recording the 'mean intensity'. The pMad intensities are plotted on different y-axes because each imaging was performed in different session upon attaining the timepoint.

Supplementary Figure S1: (A) Interleaved scatter plot showing the number of autophagic vesicles in *Atg5* overexpression (Atg5 OE) GSCs upon CQ treatment. n=20 GSCs per treatment. (B) Representative image of a cross-section of the central plane of the germarium showing eGFP fluorescence from eGFP-Atg5 (*Atg5* overexpression). eGFP fluorescence is visible in the GSC which marked by the dashed outline and bright puncta are visible in region 2. (C) Interleaved scatter plot showing the number of Ref(2)P puncta in control and *Atg5* overexpression (Atg5 OE) GSCs upon CQ treatment. n=21±1 GSCs per genotype per treatment. (D) Interleaved scatter plot showing Ref(2)P in the germarium upon CQ treatment. n=21±1 germaria per genotype per treatment. These experiments were performed twice independently, showing similar results. (E-E') Representative image showing Ref(2)P in the GSCs. The GSCs are marked by dotted outlines and the cap cells are marked by asterisks. Scale bar 10 μm. Interleaved scatter plots showing number (F) and size (G) of Ref(2)P puncta in GSCs. n=9 & 12 GSCs for control &*Atg5RNAi* respectively. Interleaved scatter plots showing the size (H) and number (I) of Ref(2)P puncta upon *Atg8a* knockdown in the cap cells and the same for size of puncta upon *Atg1* knockdown (J) in the cap cells. n=10±1 (*Atg8a RNAi*) and n=31±1 (*Atg1 RNAi*) cap cell planes/area per genotype. (K) List of all the Atgs tested for knockdown in the niche and the observation of Ref(2)P in cap cells. In all graphs blue line represents the average and error bars represent standard deviation. *$p<0.05$, **$p<0.01$, ****$p<0.0001$.

Supplementary Figure S2: (A) Graph showing change in the number of cells during aging. Average cap cell numbers are plotted as lines against the left y-axis and average GSC numbers are plotted as bars against the right y-axis. Cap cell numbers from the data set as indicated in Figure 3H with the addition of HhGal4/+ control. Error bars represent the standard error of the mean. (B) Stacked column graph showing distribution of the fraction of germaria with TUNEL-positive cap cells from the mentioned genotypes, out of all germaria assessed (cumulative) across age. n=80 germaria per genotype per time point for (A) and (B). Bar graphs showing the number of cap cells (C) and GSCs (D). Error bars represent standard deviation. Sample sizes are the number of germaria as indicated in the graphs. (E) Interleaved scatter plots showing pMad intensity in GSCs of the mentioned genotypes at the five mentioned timepoints. n=20±2 GSCs per genotype per time point.**$p<0.01$, ***$p<0.001$, ****$p<0.0001$.

Supplementary Figure S1

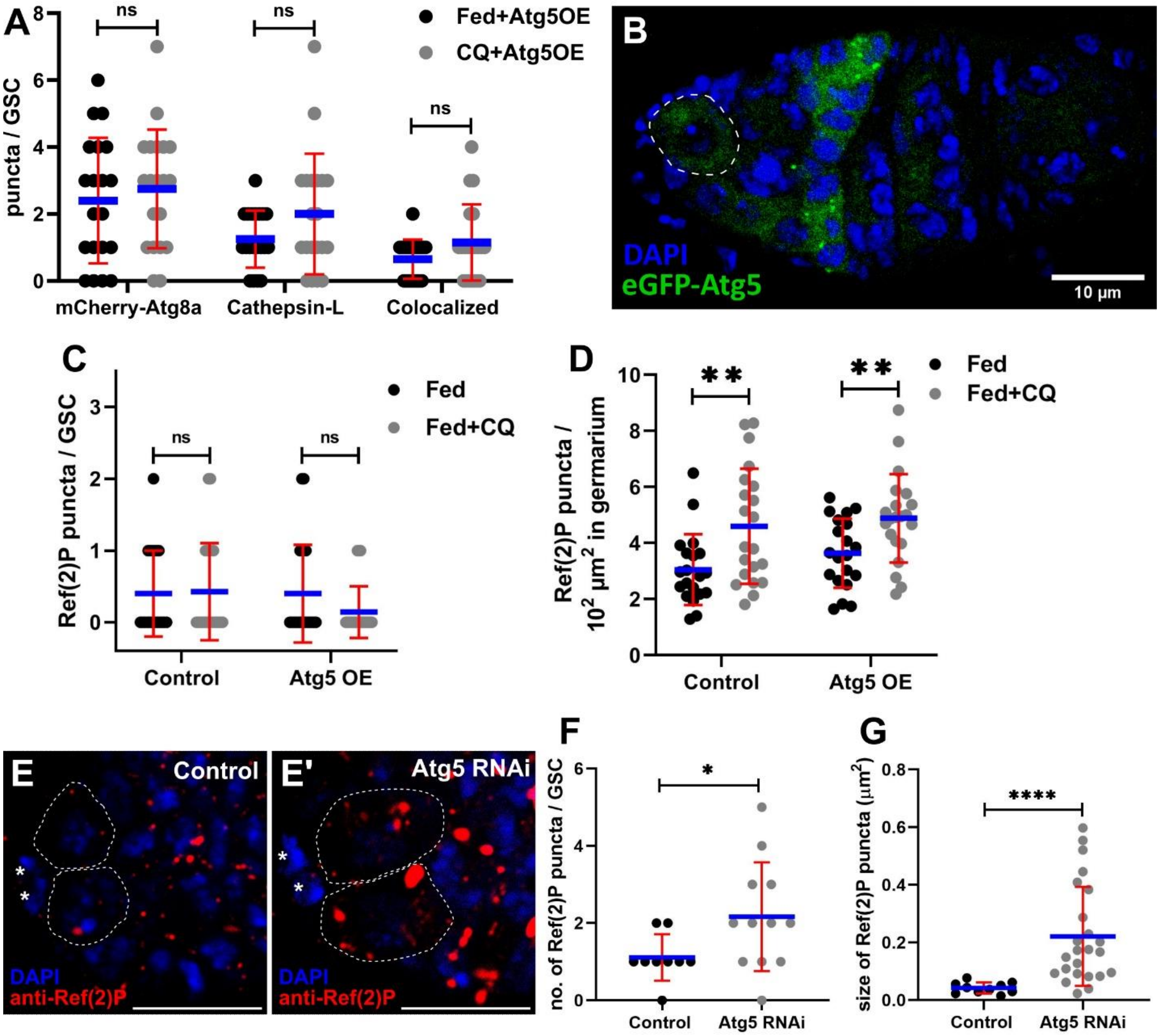

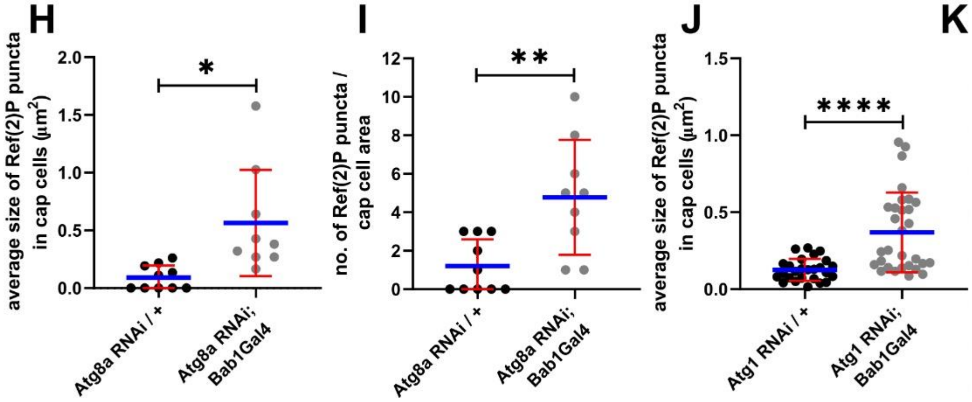

K

Atg RNAi	Ref(2)P accumulation in CC
Atg1	Yes, severe ✓
Atg5	Yes, severe ✓
Atg8a	Yes, severe ✓
Atg9	Yes, severe ✓
Atg16	Yes ✓
Atg12	No
Atg13	No

Supplementary Figure S2

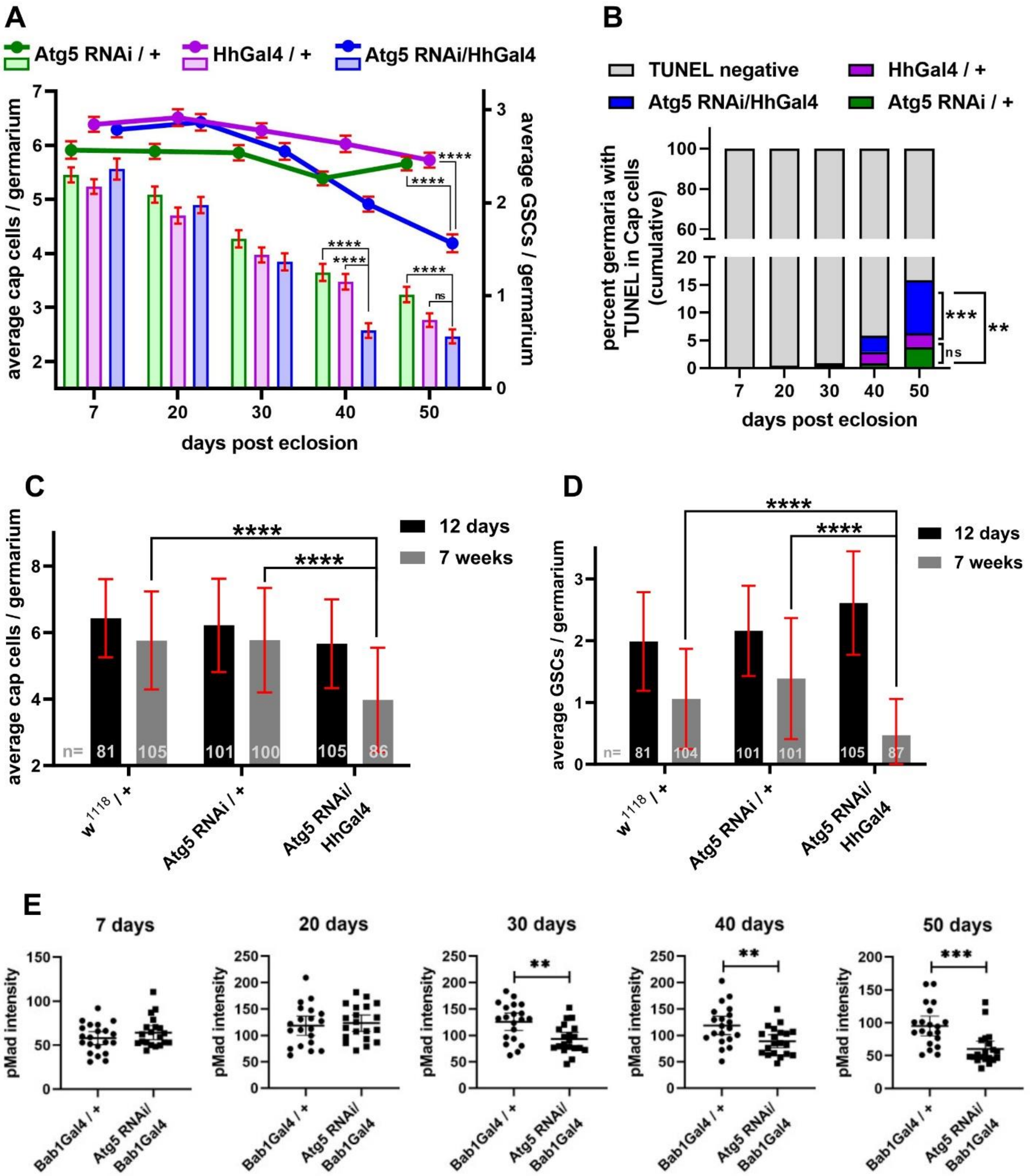

Supplement: Document S2. Article plus supplemental information [file mmc2.pdf]
